# Advancing osteoarthritis therapy with GMOCS hydrogel-loaded BMSCs-exos

**DOI:** 10.1186/s12951-024-02713-z

**Published:** 2024-08-19

**Authors:** Renyi Zhou, Jiarong Guo, Zhe Jin

**Affiliations:** Department of Orthopedics, The First Hospital of China Medical University, No. 155 Nanjing North Street, Shenyang, 110001 Liaoning Province China

**Keywords:** Osteoarthritis, Hydrogel, Exosomes, TGFB1, Nrf2 signaling, Cartilage regeneration

## Abstract

This study investigated the mechanism of the extracellular matrix-mimicking hydrogel-mediated TGFB1/Nrf2 signaling pathway in osteoarthritis using bone marrow mesenchymal stem cell-derived exosomes (BMSCs-Exos). A GMOCS-Exos hydrogel was synthesized and evaluated for its impact on chondrocyte viability and neutrophil extracellular traps (NETs) formation. In an OA rat model, GMOCS-Exos promoted cartilage regeneration and inhibited NETs formation. Transcriptome sequencing identified TGFB1 as a key gene, with GMOCS-Exos activating Nrf2 signaling through TGFB1. Depletion of TGFB1 hindered the cartilage-protective effect of GMOCS-Exos. This study sheds light on a promising therapeutic strategy for osteoarthritis through GMOCS-Exos-mediated TGFB1/Nrf2 pathway modulation.

**Supplementary Information:**

The online version contains supplementary material available at 10.1186/s12951-024-02713-z.

## Introduction

Osteoarthritis (OA) is a prevalent condition affecting joints [1, 2]. According to data from the World Health Organization, over 300 million middle-aged and elderly individuals worldwide are affected by osteoarthritis (OA) [3]. The incidence of this condition is steadily rising due to the global aging population, making it a prominent cause of disability among the elderly worldwide [4]. Osteoarthritis is characterized by several primary pathological changes, namely cartilage degeneration, osteophyte formation at the joint margins, and synovitis [5]. Extensive research has demonstrated that several factors, including genetics, age, gender, joint trauma, and mechanical stress, may be associated with the development of osteoarthritis (OA) [6–8].

In recent years, stem cell therapy has demonstrated potential in treating diverse diseases. Among these diseases, mesenchymal stem cells (MSCs) derived from various sources are considered promising cell types due to their minimal transplant rejection reactions and strong differentiation potential [9, 10]. Exosomes derived from MSCs play a crucial role in intercellular communication by transporting various bioactive molecules, including RNA, proteins, and lipids. Consequently, exosomes have emerged as a notable focus in disease treatment [11–14].

TGFB1 is a multifunctional cytokine that plays critical roles in various biological processes, such as cell growth, cell differentiation, apoptosis, immune regulation, and fibrosis [15–17]. Furthermore, the Nrf2 signaling pathway plays a pivotal role in defending against oxidative stress and regulating inflammatory responses [18–22]. Previous research has demonstrated the interaction between TGFB1 and Nrf2 in different diseases. However, their specific roles and potential therapeutic benefits in osteoarthritis (OA) have yet to be fully elucidated [23].

Neutrophils, as the predominant subset of white blood cells, not only play a role in acute inflammatory responses within the body but also participate in the pathogenesis of many chronic diseases, including osteoarthritis (OA) [24–26]. Numerous studies have demonstrated a close link between the immune infiltration of neutrophils and the development and progression of osteoarthritis (OA). Consequently, regulating these cells could offer novel treatment approaches for OA [27].

This study aims to investigate the impact of TGFB1, carried by MSCs exosomes, on neutrophil function in osteoarthritis (OA) via the Nrf2 signaling pathway. By doing so, it aims to reveal the crucial role of TGFB1 in the pathological process of OA. Through extensive research on this mechanism, we aim to offer innovative treatment strategies for OA, ultimately enhancing the management of clinical symptoms and the overall quality of life for OA patients.

## Materials and methods

### Ethics statement

Informed consent was obtained from all patients involved, and the collection and processing of clinical samples adhered strictly to ethical guidelines. All participants possess a comprehensive understanding of the research objective, and they have duly signed an informed consent form. The study has undergone review and approval by the Ethics Committee of The First Hospital of China Medical University and strictly adheres to the principles outlined in the Helsinki Declaration ([2019]2019-142-2). All animal experiments have adhered to the regulations and guidelines set forth by The First Hospital of China Medical University Animal Experiment Ethics Committee (no. 2,019,014). All experiments are conducted to minimize animal suffering [28].

### Cell isolation and culture

Our institute performed total knee arthroplasty on 10 patients with osteoarthritis (OA), with an average age of 65 years, comprising 5 males and 5 females, all without any other systemic diseases. During the surgical procedure, we isolated cartilage tissue from the medial condyle of the femur and cut it into approximately 3 × 3 mm fragments using a sterile surgical blade. The fragments were then digested in 30% pancreatin (P4201, Beyotime) for 2 min, followed by pancreatin removal and digestion with type II collagenase (17,101,015, Thermo Fisher Scientific Inc.) at a concentration of 16 mg/mL for 2 h. Chondrocytes were obtained through sieving and centrifugation, yielding primary chondrocytes. These cells were cultured in DMEM/F12 medium containing 12% FBS and 20% P/S under conditions of 37 °C and 5% CO_2_.

**Fig. 1:**
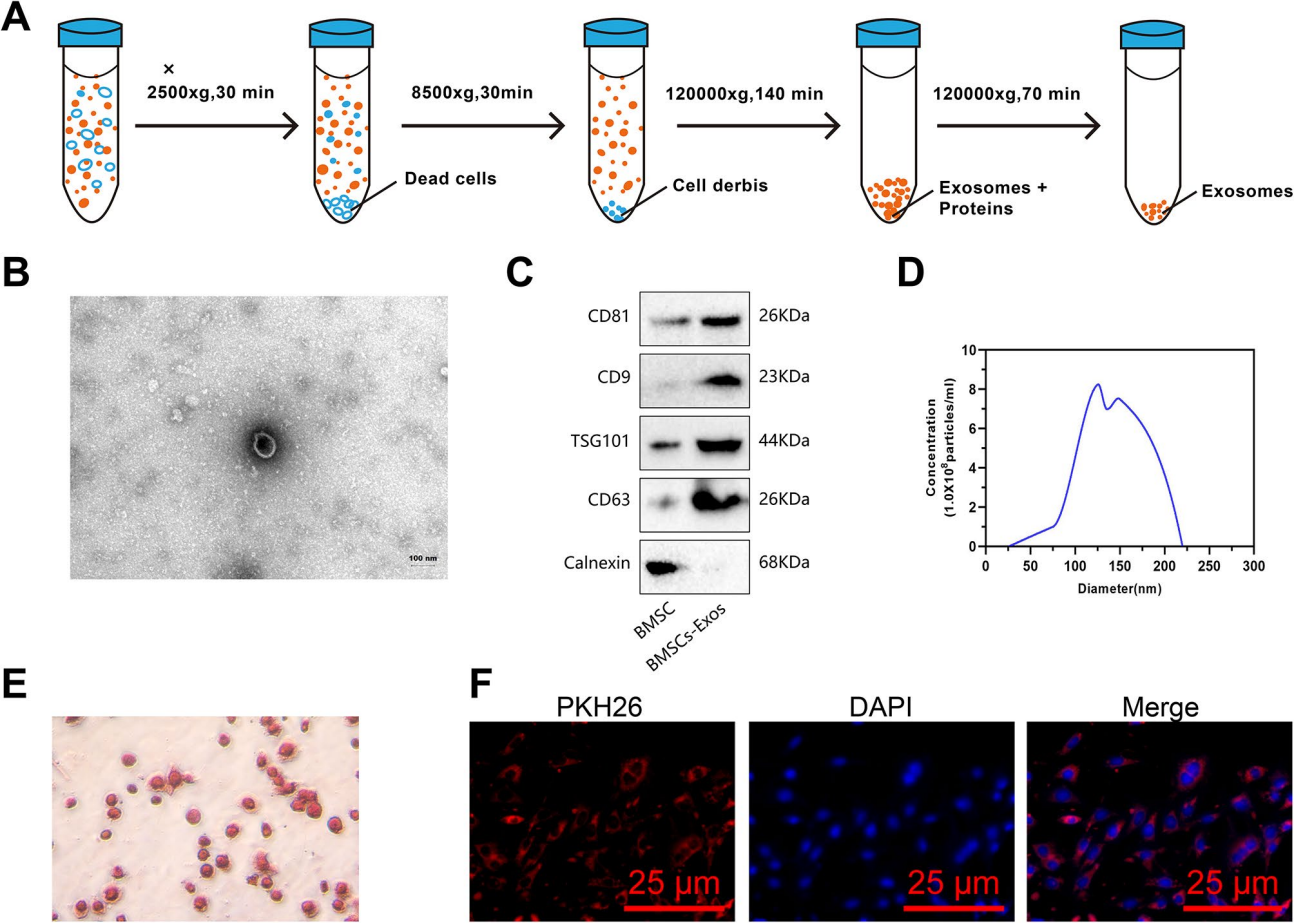
Identification of BMSCs-Exos. *Note***(A)** Schematic representation of BMSCs-Exos isolation; **(B)** Transmission electron microscopy images of BMSCs-Exos, with a scale bar of 200 nm on the left panel and 50 nm on the right panel; **(C)** Western blot analysis of CD81, CD9, TSG101, CD63, and Calnexin expression in BMSCs-Exos; **(D)** Dynamic light scattering analysis of BMSCs-Exos particle size; **(E)** Cell rotation images of purified SF neutrophils, with a scale bar of 50 μm; **(F)** Fluorescence microscopy observation of neutrophil uptake of BMSCs-Exos, with a scale bar of 50 μm. Cell experiments were repeated three times

Synovial fluid (SF) was collected during joint replacement surgery in OA patients, and neutral granulocytes were isolated from SF using Ficoll^®^ 400 lymphocyte subset separation reagent (341,691, Sigma-Aldrich, Shanghai, China). The purity of the isolated neutral granulocytes from SF was assessed to be > 97% based on May-Grunwald Giemsa staining (YB160462, Shanghai Yubo Biotech Co., Ltd., Shanghai, China) (Fig. 1E).

The separated neutral granulocytes were cultured in a confocal culture dish and stimulated with PMA (100 nM, P1585, Sigma-Aldrich) for 4 h at 37 °C to promote the formation of NETs. The cells were fixed in 4% paraformaldehyde (P0099, Beyotime) for 10 min and permeabilized with Triton×100 (93,443, Sigma-Aldrich) for 5 min. Cell washing was performed with Tris-buffered saline containing 0.1% TWEEN^®^ 20 (ZY-6,191,005, Shanghai Zeye Biotech Co., Ltd., Shanghai, China), followed by cell blocking with 5% bovine serum albumin and staining with rabbit monoclonal Elastase antibody (PA5-88916, 1:50, Thermo Fisher Scientific Inc.) and Alexa Fluor 488-conjugated goat anti-rabbit IgG secondary antibody (A32731, 1:2000, Thermo Fisher Scientific Inc.). DNA was stained with DAPI (C1005, Beyotime), and observation was performed under a confocal fluorescence microscope (IX73, Wuhan Jezhi Technology Co., Ltd., Wuhan, China) [29–31].

Human bone marrow mesenchymal stem cells (BMSCs) were purchased from Thermo Fisher Scientific Inc. All cells were cultured in DMEM medium supplemented with 10% FBS and 1% P/S under conditions of 37 °C and 5% CO_2_. Upon reaching 70–80% confluency on the culture flask, the medium was replaced with exosome-free DMEM, and the cells were further incubated [32].

The morphological characteristics of BMSCs were evaluated under an optical microscope (TS2009, Henan Yunfei Technology Development Co., Ltd., Henan, China). Following osteogenic, chondrogenic, and adipogenic induction of BMSCs, alizarin red staining was used to confirm bone formation, oil red O staining was utilized to validate fat formation, and Alcian blue staining was applied to detect cartilage formation. Furthermore, flow cytometry was used to identify the negative and positive surface markers of BMSCs [28].

### BMSC osteogenic induction and alizarin red staining

The cells were cultured in an osteogenic induction medium containing 10% FBS, 2% P/S, 0.01 µmol/L dexamethasone (ST1254, Beyotime), 10 mmol/L β-glycerophosphate (IG1340, Solarbio), and 50 mg/L ascorbic acid (A8100, Solarbio) per 100 mL. Alizarin red staining was performed. Cells were fixed with 4% paraformaldehyde for 15 min, followed by adding 2 mL of 0.2% alizarin red staining solution (T4121, Beyotime) to each well for 30 min. After washing away the residual dye, the staining results were observed under a microscope [33].

### Chondrogenesis induction and alcian blue staining of BMSC

The cells were cultured in a chondrogenic induction medium containing 10% FBS, 1% P/S, 100 nM dexamethasone, 50 µg/mL L-ascorbic acid, 100 µg/mL sodium pyruvate (C0331, Beyotime), 40 µg/mL proline (ST1500, Beyotime), 50 mg/mL insulin-transferrin-selenium (I1884, Sigma-Aldrich), and 10 ng/mL transforming growth factor-beta (TGF-β) and stained with Alcian blue. The cells were fixed with 4% paraformaldehyde for 15 min, stained with Alcian blue staining solution (C0155M, Beyotime) for 30 min, rinsed to remove excess dye, and observed under a microscope for the staining results [33].

### BMSC adipogenic induction and oil red O staining

The cells were cultured in an adipogenic induction medium containing 10% FBS, 1% P/S, 10 µg/mL insulin (P3376, Beyotime), 1 µM dexamethasone, 0.5 mM IBMX (SC0195, Beyotime), and 0.1 mM indomethacin (SI9020, Solarbio), followed by Oil Red O staining. The cells were fixed with 10% neutral formalin for 30 min, stained with 0.5% Oil Red O working solution (C0157S, Beyotime) for 1 h for dark staining, rinsed with isopropanol (563,935, Sigma-Aldrich) to remove excess dye, and then observed under a microscope for the staining results [33].

### Flow cytometry analysis

To analyze the surface markers of BMSCs, we utilized the following antibodies: CD29 (ab193591, 1:500, Abcam, Boston, MA, USA), CD44 (130-113-904, 1:50, Miltenyi Biotec, Germany), CD90 (ab307736, 1:500, Abcam), CD105 (ab114052, 1:200, Abcam), CD11b (ab8878, 1:1000, Abcam), CD45 (ab303670, 1:500, Abcam), CD31 (ab7388, 1:100, Abcam), and CD34 (ab81289, 1:50, Abcam). Flow cytometric analysis was conducted using a flow cytometer (Attune NxT, Thermo Fisher Scientific Inc.) [28].

### Extraction and identification of BMSCs-exos

BMSCs were cultured in serum-free DMEM for 2 days. The culture medium was collected and centrifuged at 2500 g and 8500 g for 30 min to eliminate dead cells and cell debris. The supernatant was centrifuged at 120,000 g for 140 min. After washing with PBS, the pellet was resuspended in 100 µL of PBS. The extracellular vesicles were obtained by centrifugation at 120,000 g for 70 min (Fig. 1A). The morphology of exosomes was observed using a high-resolution transmission electron microscope (Talos L120C TEM, Thermo Fisher Scientific Inc.). Through Western blot analysis, surface markers CD9, CD63, CD81, TSG101, and Calnexin were detected in exosomes. Additionally, the size of exosomes was determined using a nanoparticle tracking analyzer (NanoSight NS300, Shanghai SIBATA Scientific Technology Co., Ltd., Shanghai, China) [32, 34].

### Preparation of cell-derived extracellular matrix-mimicking hydrogel loaded with BMSCs-exos

The construction method for the extracellular vesicle hydrogel system is as described above [35]. Chondroitin sulfate (CS, BCN1312, BioKorea, Chengdu, China) should be dissolved in distilled water with stirring until fully dissolved (5% (w/v)). Then, the CS solution should add 1.93 g of sodium metaperiodate (311,448, Sigma-Aldrich). Allow the reaction to occur in the dark for 12 h. Next, transfer the solution into a dialysis bag with a molecular weight cut-off of 3500 Dalton and immerse it in distilled water at 50 °C for 24 h. The dialysis solution was freeze-dried in a freeze-dryer for 6 days to obtain oxidized chondroitin sulfate (OCS). To prepare a solution of GMOCS, combine a 12.5% (w/v) solution of methyl acryloyl gelatin (GM, 934,798, Sigma-Aldrich) with PBS containing OCS. The solution is dissolved in a water bath at 70 °C until no residues or dense foam exists. Subsequently, it is promptly sterilized using a 0.22 μm filter. According to the manufacturer’s instructions, the GMOCS solution, Exos, and photosensitizer 2959 (0.5% w/v, 106797-53-9, Suzhou Sosen New Material Technology Co., Ltd., Suzhou, China) should be mixed in a centrifuge tube. Then, the tube should be rotated for 3 min to ensure the even distribution of Exos in the solution. GMOCS-Exos, a water gel-based material, was synthesized through crosslinking using ultraviolet radiation (6.9 mW/cm2, 360–480 nm) for 10 s [35].

### Characterization of GMOCS-Exos

The iodometric titration method determined the degree of oxidation (DO) in oxidized CS. The molecular weight (MW) of GM and OCS was analyzed using the Agilent PL-GPC50/Agilent1260 room-temperature gel permeation chromatography instrument manufactured by Beijing Polytec Technology Co., Ltd. Hydrogels’ internal morphology and structure were examined in Shanghai, China, using TESCAN’s field emission scanning electron microscope. The chemical composition of the water gel was analyzed using a Fourier Transform Infrared spectrometer, specifically the Nicolet 6700 model. Moreover, the storage modulus (G’) and loss modulus (G″) of GMOCS hydrogels were measured using Anton Paar’s Physica MCR301 rheometer. Dynamic oscillatory frequency sweeps (0–1.5 Hz) were then conducted at a fixed strain of 10%. The compressive strength of the hydrogel was determined using the Q800 DMA tester from TA Instruments, which was then used to calculate the stress-strain curve [35].

### Extracellular vesicle uptake assay

Following the manufacturer’s protocol for exosome uptake study, exosomes were labeled with red fluorescent dye PKH67 (EK26002, from Nanjing Yike Biotechnology Co., Ltd.). A mixture of 100 µL exosome suspension and 10 µL PKH67 (diluted in diluent C at a ratio of 1:25) was incubated at 10 °C for 37 min. The staining was stopped with 1 mL of 0.5% BSA (P0007, Beyotime), and exosomes were re-extracted by ultracentrifugation at 110,000 × g for 70 min. Chondrocytes were co-cultured overnight with PKH67-labeled exosomes (10 µg/mL). To confirm the cellular uptake of exosomes in hydrogels, chondrocytes and neutrophils were co-cultured with GMOCS-Exos. After 24 h, the cells were fixed in 20% paraformaldehyde for 33 min and nuclei were stained with DAPI-containing anti-quenching mounting medium (S2100, Solarbio). Images were acquired using a confocal fluorescence microscope [36].

### Extracorporeal biocompatibility assessment

In vitro biocompatibility assessments were conducted, including cell viability, proliferation, and adhesion experiments. For live/dead cell staining, chondrocytes at a density of 1 × 10^6^ cells were seeded in a 12-well culture plate and co-incubated with each sample for 24 h. A live/dead cell staining solution was prepared in a ratio of 1 mL: 3 µL: 5 µL (PBS: calcein-AM: PI) and added to each group, followed by an incubation at 30 °C for 37 min. After co-culturing 1 × 10^6^ chondrocytes with the samples for 1, 3, and 7 days, 100 µL of CCK-8 solution (C0037, Beyotime) was added to each well and incubated for 2 h. The supernatant (100 µL) was transferred to a 96-well plate, and the absorbance was measured at 450 nm using a microplate reader (BioTek Synergy Neo2 Hybrid, Agilent Technologies (China) Co., Ltd., Beijing, China). Cell adhesion to hydrogels was evaluated by culturing cells at a density of 1 × 10^5^ cells per well for 3 days, fixing with 4% paraformaldehyde, staining with Actin-Tracker Green-488 (C2201S, Beyotime) and Hoechst (C1011, Beyotime), and observing cellular morphology using a confocal microscope [35].

### Cell infection

Negative control (NC) shRNA, TGFB1 shRNA (sh-TGFB1), and Nrf2 shRNA (sh-Nrf2) lentiviruses were obtained from Genepharma in Shanghai, China. Follow the cell infection protocol provided by the supplier. The cells should be incubated in the supernatant of the reverse transcription virus containing 5 µg/mL of polyamine (TR1003, Sigma-Aldrich) for 24 h. Cells were treated with puromycin (540,411, Sigma-Aldrich) at a concentration of 2.5 µg/mL in the culture medium 48 h after infection. The sequences of sh-Nrf2, sh-TGFB1, and sh-NC are as follows: sh-Nrf2 sequence is 5′-GGAAAGACAAGAACAACTCCA-3′; sh-TGFB1 sequence is 5′-GCAGAGTACACACAGCATATA-3′; and sh-NC sequence is 5′-CCTAAGGTTAAGTCGCCCTCG-3′ [37].

### Establishment and treatment of rat models of osteoarthritis

Male Sprague-Dawley rats weighing 300–350 g (approximately 12 weeks old, Beijing VENTILIHUA Experimental Animal Technology Co., Ltd., China) were used in this study. The osteoarthritis (OA) model was induced by complete transection of the medial collateral ligament and medial meniscus without damaging the tibial surface, cutting the meniscus at its narrowest point, and transecting the anterior cruciate ligament. The rats were anesthetized using an animal anesthesia machine (R620-S1, Shenzhen Ruowode Life Technology Co., Ltd., China).

The specific surgical procedures for transecting the medial collateral ligament and medial meniscus can be found in the references [38, 39]. Postoperatively, approximately 15 µl of GMOCS-Exos mixture were injected into the joints, followed by rapid in situ crosslinking under ultraviolet irradiation. Sprague-Dawley rats were randomly divided into 9 groups: (1) Sham group (sham surgery, 5 rats with 10 knees, *n* = 10); (2) OA group (rats underwent surgery with joint cavity injection of saline after the procedure, 5 rats with 10 knees, *n* = 10); (3) GM group (OA rats received GM injection as a positive control, 5 rats with 10 knees, *n* = 10); (4) GMOCS group (OA rats received GMOCS injection, 5 rats with 10 knees, *n* = 10); (5) Exos group (OA rats received Exos injection, 5 rats with 10 knees, *n* = 10); (6) GMOCS-Exos group (OA rats received GMOCS-Exos injection, 5 rats with 10 knees, *n* = 10); (7) GMOCS-Exos + sh-NC group (OA rats received GMOCS-Exos hydrogel loaded with BMSCs-Exos infected with sh-NC, 5 rats with 10 knees, *n* = 10); (8) GMOCS-Exos + sh-Nrf2 group (OA rats received GMOCS hydrogel loaded with BMSCs-Exos infected with sh-Nrf2, 5 rats with 10 knees, *n* = 10); (9) GMOCS-Exos + sh-TGFB1 group (OA rats received GMOCS hydrogel loaded with BMSCs-Exos infected with sh-TGFB1, 5 rats with 10 knees, *n* = 10).

PKH26 was utilized for labeling exosomes, which were then incorporated into GMOCS hydrogel. The PKH26-labeled exosomes and GMOCS-Exos were locally injected into the joint cavity using a pre-chilled Hamilton syringe (Hamilton 800, Conlin Technology Co., Ltd., Beijing, China). Imaging of PKH26 intensity and distribution in the knee joint was performed on days 1, 4, and 7 using an in vivo imaging system for small animals (IVIS, IVIS^®^ Lumina LT Series III, PuHua Quantum Technology Co., Ltd., Beijing, China). Eight weeks post-surgery, euthanasia was carried out by cervical dislocation, and samples of the knee joint and synovial fluid were collected [35, 38, 39].

### Hemolysis test and degradation research

Whole blood from rats was centrifuged at 10,000 × g for 10 min at 4 °C to separate red blood cells. The red blood cells were washed three times with physiological saline and diluted to a 5% concentration. The prepared hydrogel samples were added to the red blood cell suspension and incubated at 37 °C for 24 h to study hemolysis changes. PBS and Triton X-100 were used as negative and positive controls, respectively. After centrifugation, the supernatant was transferred to a new 96-well plate. Absorbance at 540 nm was measured using a UV-visible spectrophotometer to determine the OD value of the supernatant. The percentage of red blood cell hemolysis was calculated using the formula: RBC hemolysis = 100% × (ODsample – ODPBS) / (ODtriton – ODPBS) [35, 40].

To simulate in situ degradation conditions, GMOCS hydrogel was incubated at 37 °C in 5 mL of PBS. The weight of the hydrogel was measured at different time points after removing excess moisture with filter paper. The degradation of the hydrogel was calculated by dividing the final weight of the hydrogel by the initial weight of the hydrogel [35].

### RT-qPCR

Total RNA was extracted from tissues and cultured cells using TRIzol reagent (Thermo Fisher Scientific, catalog number 15,596,026) according to the manufacturer’s instructions. Reverse transcription of the extracted RNA into cDNA was performed using the PrimeScript™ RT reagent Kit with gDNA Eraser reverse transcription kit (TaKaRa Bio, Beijing, China, catalog number: RR047A). RT-qPCR was conducted using TB Green^®^ Premix Ex Taq™ (Tli RNaseH Plus) (Cat. No. RR420A, TaKaRa Bio (Beijing) Co., Ltd.) and gene-specific primers at a final concentration of 0.3 nM. The primer is shown in (Table S1).

The relative quantification method (2^−ΔΔCt^) was employed to calculate the relative transcription levels of the target gene, with β-actin expression serving as the internal reference. Three replicate wells are set up for each sample, and the experiment is repeated three times [35].

### Western blot

Proteins were extracted from tissues, BMSCs-Exos, or cells using RIPA buffer (P0013B, Beyotime) supplemented with phenylmethanesulfonyl fluoride (PMSF, ST505, Beyotime). The protein concentration was determined using the BCA Protein Assay Kit (P0011, Beyotime). The protein sample (10 µg) was separated using 10% SDS-PAGE (P0690, Beyotime) and then transferred onto a nitrocellulose membrane (FFN02, Beyotime) following standard protocols.

The samples were blocked with 5% skim milk in PBST for 1 h. After that, they were incubated overnight at 4 °C with primary antibodies, namely CD9 (1:1000, ab307085, Abcam), CD63 (1:200, ab216130, Abcam), CD81 (1:1000, ab109201, Abcam), TSG101 (1:1000, ab133586, Abcam), Calnexin (1:200, ab227310, Abcam), COL2A1 (1:1000, ab307674, Abcam), SOX9 (1:1000, ab185966, Abcam), MMP13 (1:3000, ab39012, Abcam), Nrf2 (1:1000, ab313825, Abcam), NQO-1 (1:10,000, ab80588, Abcam), HO-1 (1:2000, ab189491, Abcam), TGFB1 (1:1000, ab215715, Abcam), and GAPDH (1:1000, ab125247, Abcam). Subsequently, the secondary antibody labeled with peroxidase (1:1000, ab6721, Abcam) was incubated for 1 h.

Finally, all protein bands were observed, captured, and analyzed using a gel imaging system (Syngene G: BOX F3, Antpedia Technology Development Co., Ltd, Beijing, China) and ImageJ software (NIH, Bethesda, MD, USA). The relative content of the target protein is represented by dividing the grayscale value of the target protein band by the grayscale value of the internal reference protein band [28]. The cell experiment is repeated three times.

### Cytology of organisms

Rat articular cartilage specimens fixed in paraformaldehyde were decalcified in 10% EDTA (pH 7.4) for 21 days, followed by embedding in paraffin and sectioning at a thickness of 5 μm. Serial sections were taken from both compartments at 200 μm intervals. Selected sections underwent deparaffinization in xylene, followed by ethanol gradient washes and hydration. Histological staining was performed using hematoxylin and eosin (H&E) according to the reference [41]. Chondrocyte nuclei appeared blue, while other tissues stained pink.

The staining procedure with Toluidine Blue (TB) was conducted following the guidelines provided in PMID: 26,683,663. Under light microscopy observation, the cartilage exhibited a bluish-purple color, while the background appeared light blue.

The Safranin O-Fast Green (SO-FG) staining procedure followed the method described in PMID: 31,511,005. Under light microscopy observation, the cartilage appeared red, while the background exhibited a green hue.

Osteoarthritis severity was evaluated using the Osteoarthritis Research Society International (OARSI). The OARSI score consists of eight levels ranging from 0 to 6, each indicating different degrees of cartilage damage. In this scale, 0 signifies cartilage in normal condition without any damage, 0.5 indicates loss of Safranin O staining without noticeable structural changes, 1 represents minor fibrosis, 2 indicates vertical cartilage damage restricted to the superficial layer, 3 represents vertical damage not exceeding 25% of the cartilage surface, 4 denotes vertical damage accounting for 25–50% of the cartilage surface, 5 denotes vertical damage accounting for 50–75% of the cartilage surface, and 6 signifies vertical damage accounting for over 75% of the cartilage surface. The higher the level, the more severe the osteoarthritis [42].

### ROS detection in neutrophils

The production of intracellular reactive oxygen species (ROS) could be detected using the DHE fluorescent dye (ID3560, Solarbio). Cells were cultured in Hank’s Balanced Salt Solution (HBSS, C0219, Solarbio) supplemented with CaCl2 and MgCl2. Subsequently, 10 µmol/L of DHE was added. The cells were incubated at 37 °C in a light-controlled environment for 30 min. The cells should be washed with HBSS and loaded onto a microscopy slide using a mounting medium containing DAPI. Finally, images of the cells should be captured using a fluorescence microscope. The intracellular reactive oxygen species (ROS) level was measured using a flow cytometer with an excitation wavelength of 535 nm and an emission wavelength of 610 nm [43]. All experiments were repeated three times.

### Establishment of the co-culture system of GMOCS-Exos/neutrophils/chondrocytes

To investigate the impact of systemic neutrophil extracellular traps (NETs) on chondrocytes, a coculture system of GMOCS-Exos/neutrophils/chondrocytes was established in a Transwell chamber. Prior to the experiment, chondrocytes were treated with IL-1β (10 ng/mL) for 24 h, while neutrophils were stimulated with PMA (100 nM) for 4 h to induce NET formation. GMOCS-Exos were cocultured with 1 × 10_5_ neutrophils in the lower chamber, and 1 × 10_4_ chondrocytes were placed in the upper chamber. A polycarbonate membrane with a pore size of 1.0 μm (10,418,712, Shanghai Bestet Biotechnology Co., Ltd., Shanghai, China, https://bestest.cn.china.cn/) separated the two layers, allowing free passage of cytokines without affecting cell interactions [35].

### Assessment of proliferation, apoptosis and migration of chondrocytes

Proliferation of cartilage cells is assessed using the EdU assay kit. By establishing a co-culture system comprising GMOCS-Exos, neutrophils, and chondrocytes, it becomes possible to assess the proliferative potential of the chondrocytes. Chondrocytes were cultured in a medium containing 24 µM of EdU for 10 h. Follow the manufacturer’s instructions to evaluate the proliferation rate and employ the BeyoClick™ EdU-594 Cell Proliferation Assay Kit (C0078S, Beyotime). A fluorescence microscope is utilized to analyze the cell proliferation in each group [44].

The evaluation of chondrocyte apoptosis involved investigating the impact of GMOCS-Exos on chondrocyte apoptosis using the TUNEL assay kit. Chondrocytes in each group were stained using the TUNEL bright green apoptosis detection kit (C1086, Beyotime), following the manufacturer’s instructions. Subsequently, they were examined and photographed under a fluorescence microscope [44].

Migration assessment of chondrocytes was conducted using the Transwell assay and cell scratch wound healing assay. Chondrocytes were seeded at a density of 100% in a 6-well plate for the cell scratch assay. Create a scratch on the cell layer using a sterile pipette tip with a volume of 200 µL. Cells should be observed and photographed at 0, 24, and 48-hour intervals. Before conducting the scratch assay, cells were treated with colcemid (1 µg/mL) for 1 h to eliminate any potential effects of cell proliferation [44].

In Transwell assays, chondrocytes were seeded in the upper chamber and incubated at 37 °C for 24 h. The upper chamber of the Transwell was subsequently fixed with 20% paraformaldehyde (PFA) for 4 min. Afterward, the upper surface of the chamber was wiped to eliminate non-migrated cells. Following a 30-minute incubation in 0.5% Crystal Violet (C0121, Beyotime), wash the chamber three times with a PBS solution. Next, a microscope was utilized to examine the migratory behavior of chondrocytes in each experimental group [44].

### Sequencing and preliminary data processing

RNA extraction was performed using TRIzol reagent following the manufacturer’s instructions. After precipitating the RNA, dissolve it in 1.5 mL of DEPC-treated water in a centrifuge tube. The concentration and purity of RNA were measured using NanoDrop, ensuring that the 260/280 ratio was between 1.8 and 2.1. To ensure RNA integrity, evaluate it further using the Agilent Bioanalyzer and verify that the RNA Integrity Number (RIN) value exceeds 7. It is recommended that rRNA depletion kits be utilized to remove rRNA from the samples. Libraries were prepared using NEB or Illumina library preparation kits. The RNA was randomly fragmented, and sequencing adapters were ligated to the fragments. The quality and size of the library products can be verified using Qubit and Agilent Bioanalyzer.

Select the suitable Illumina HiSeq or NovaSeq platform for high-throughput sequencing, considering the quality of the library and research objectives. Following sequencing, employ FastQC to assess the quality of the unprocessed sequencing data, confirming that the Q30 value exceeds 90%. Trim Galore or Trimmomatic software could be employed for quality control of reads and removing low-quality reads and Illumina adapters. The processed reads should be aligned with the reference genome using HISAT2 or STAR software, with a mapping efficiency of at least 90% [45].

### Differential expression analysis

Align the .bam files using STAR and convert them into .counts files. Differential analysis was conducted using the edgeR package. A |log2FoldChange|>1 and FDR < 0.05 threshold was applied to identify differentially expressed genes [45].

### Cartilage allograft cultivation

The cartilage tissue was rinsed with cold PBS three times. 5 × 5 × 5 mm^3^ cartilage explants were cultivated in a 37 °C, 5% CO_2_ incubator with DMEM/F12 medium supplemented with 10% fetal bovine serum, 1% penicillin/streptomycin, and human IL-1β (10 ng/mL). The cartilage grafts from the same patient were treated with or without ECH-Exos for 72 h. Subsequently, they were fixed in 4% paraformaldehyde at 4 °C for 24 h for immunohistochemical analysis [46].

### Immunohistochemistry

EDTA at a pH of 9.0 was used for antigen retrieval on dewaxed slices. The slices were immersed in a 3% hydrogen peroxide solution and incubated at room temperature in the dark for 25 min, followed by blocking with 3% BSA at room temperature for 30 min. Subsequently, the slices were incubated overnight at 4 °C with primary antibodies against Aggrecan (PA1-1746, 1:50, Thermo Fisher Scientific Inc.) and MMP13 (ab219620, 1:2000, abcam), then for 60 min at room temperature the next day. After washing, DAB-horseradish peroxidase substrate and hematoxylin solution were added. The stained samples were observed under a microscope using Image-Pro Plus version 5.0 (Media Cybernetics, Inc. USA), and the number of positively stained cells was quantified [47].

### Statistical analysis

GraphPad Prism 8 (version 8.0.2.263, GraphPad Software, USA) and R software v4.2.1 (R Foundation for Statistical Computing, Vienna, Austria) were used for statistical analysis. The measurement data was represented as Mean ± SD (mean plus/minus standard deviation). Unpaired t-tests were used to compare the differences between the two groups, whereas one-way ANOVA was used to compare the differences among multiple groups. To assess the homogeneity of variance, Levene’s method could be used. If the variances are homogeneous, pairwise comparisons could be performed using Dunnett’s t-test and LSD-t-test. When variances are not homogeneous, use Dunnett’s T3 test. A significance level of *P* < 0.05 demonstrates that the observed difference is statistically significant.

## Results

### Characterization and internalization of BMSC-derived exosomes by neutrophils

Recently, exosomes derived from BMSCs have been recognized as a new cell-free therapeutic platform for various diseases. They exhibit therapeutic effects such as promoting regeneration and modulating immune responses [48]. Firstly, we characterized the properties of BMSCs. When viewed under an optical microscope, BMSCs exhibited a spindle-shaped morphology (Fig. S1A). Additionally, BMSCs successfully underwent adipogenic, osteogenic, and chondrogenic differentiation processes (Fig. S1B-D).

Flow cytometry analysis revealed high expression levels of positive surface markers CD29, CD90, CD44, and CD105 on BMSCs, while negative surface markers CD11b, CD45, CD31, and CD34 were not expressed (Fig. S1E), confirming successful identification of BMSCs.

To fully characterize the exosome particles derived from BMSCs, we utilized transmission electron microscopy, western blot, and particle size analysis. Transmission electron microscopy revealed that BMSCs-Exos exhibited a typical cup-shaped or spherical morphology characterized by lipid membrane structures (Fig. 1B). Western blot analysis revealed the presence of positive characteristic surface markers (CD81, CD9, TSG101, and CD63) in BMSCs-Exos, while Calnexin was not detected (Fig. 1C). Particle size measurements demonstrate that the particles have an estimated size ranging from 50 to 200 nm (Fig. 1D). The results above demonstrate the successful extraction of exosomes derived from BMSCs.

Neutrophils have been demonstrated as among the first immune cells to infiltrate the synovium in the development of osteoarthritis. Furthermore, they are implicated in inflammation and the progression of the disease [49].

We experimented to investigate the effects of BMSCs-Exos on neutrophils. To do this, we labeled the exosomes with PKH26 and observed the presence of PKH67-labeled (red fluorescence) BMSCs-Exos surrounding the neutrophil nuclei (Fig. 1F). This result confirmed that the neutrophils had internalized the BMSCs-Exos.

The results above indicate the successful extraction of exosomes derived from BMSCs and the internalization of BMSCs-Exos by neutrophils.

### Preparation and characterization of GMOCS-Exos hydrogel mimicking the extracellular matrix

**Fig. 2:**
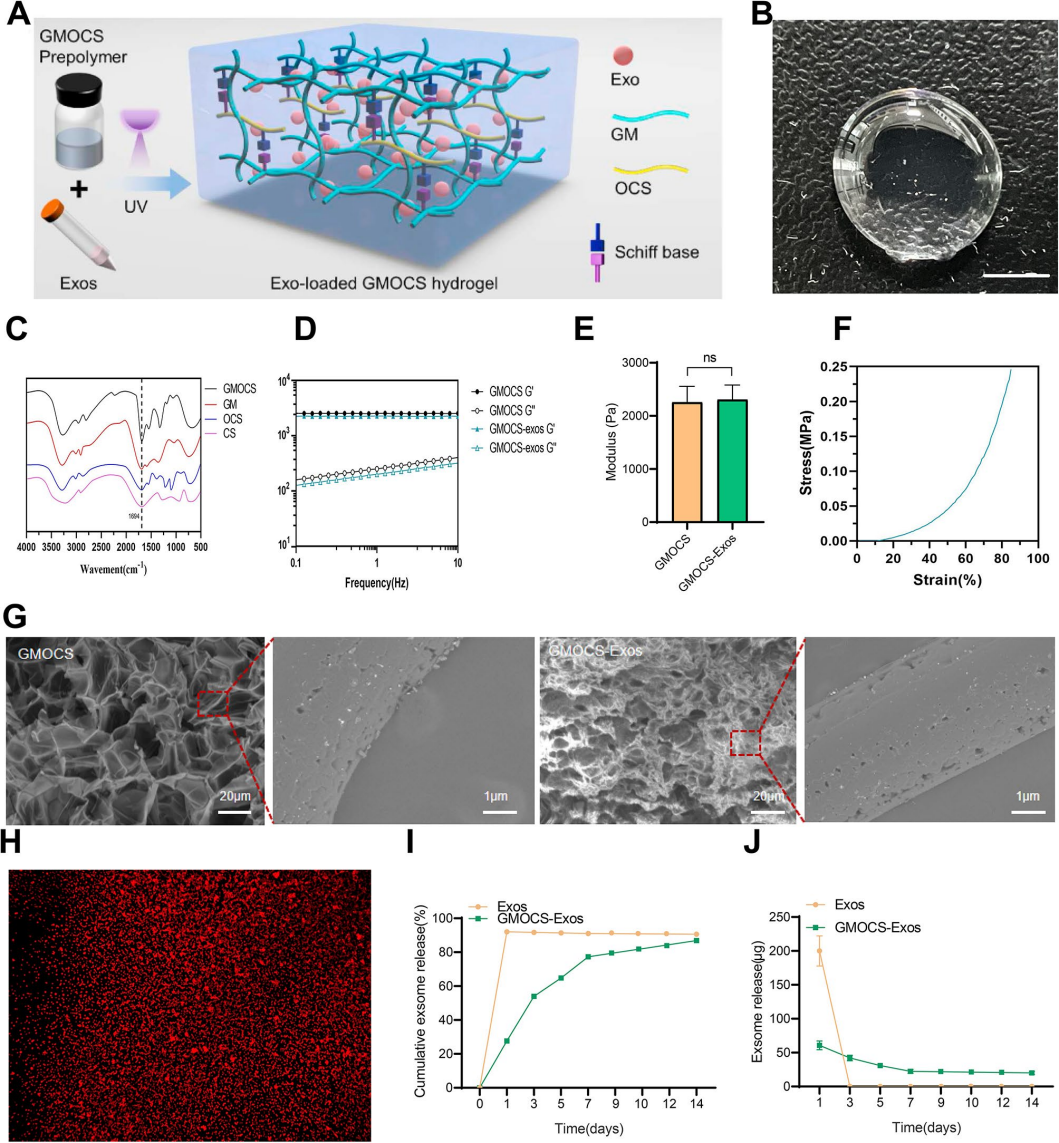
Characteristics of GMOCS hydrogel loaded with BMSCs-Exos. *Note***(A)** Schematic representation of the synthesis and chemical structure of GMOCS hydrogel; **(B)** Photograph of GMOCS-Exos hydrogel; **(C)** FTIR spectra of GM, CS, OCS, and GMOCS; **(D)** Rheological analysis of GMOCS hydrogel with or without Exos; **(E)** Average storage modulus of GMOCS and GMOCS-Exos; **(F)** Representative stress-strain curves of GMOCS hydrogel; **(G)** SEM images of GMOCS hydrogel with or without Exos, with white arrows indicating Exos loaded on the hydrogel, scale bar = 20 μm–1 μm; **(H)** Three-dimensional images of PKH26-labeled Exos in GMOCS hydrogel; **(I)** Cumulative release curve of Exos over 14 days; **(J)** Daily release curve of Exos. Data are presented as mean ± standard deviation. Ns indicates statistically not significant. Cell experiments were repeated three times

The synthesis metabolism of chondrocytes could be enhanced by inhibiting inflammation through the formation of GMOCS hydrogel, which is achieved by introducing chondroitin sulfate (OCS) into methacrylate gelatin (GM) [35]. Therefore, we loaded BMSCs-Exos into GMOCS to generate GMOCS-Exos. Figure 2A-B illustrates the process of preparation and provides images of GMOCS-Exos. GMOCS and Exos are combined and crosslinked using a photosensitizer under ultraviolet radiation to produce GMOCS-Exos hydrogel. In this process, sodium periodate is the oxidizing agent that converts the hydroxyl groups on the CS polysaccharide backbone to aldehyde groups. FTIR analysis confirmed the successful synthesis of OCS. Compared to CS, a new peak at 1694 cm − 1 appeared in the OCS spectrum (Fig. 2C).

The rheological performance tests, as shown in Fig. 2D, indicate that both GMOCS and GMOCS-Exos exhibit a higher storage modulus (G’) compared to the loss modulus (G″), which suggests that they possess favorable stability and viscoelastic properties. Furthermore, there was no notable disparity in the average storage modulus between GMOCS and GMOCS-Exos (Fig. 2E), implying that the inclusion of Exos had minimal impact on the mechanical characteristics of GMOCS. The stress-strain curve of GMOCS reveals that at 88% strain, the hydrogel exhibits a compressive strength of 0.25 MPa (Fig. 2F), meeting the compressive strength criteria for bone tissue (0.15 ~ 13.7 MPa) [50].

Additionally, scanning electron microscopy (SEM) imaging reveals a porous network structure in the hydrogel, and the internal surface of the GMOCS hydrogel shows the binding of Exos (Fig. 2G). Immunofluorescence imaging was employed to unveil the three-dimensional (3D) spatial distribution of Exos within the hydrogel. This information is illustrated in Fig. 2H. Importantly, we observed that the release of Exos from the GMOCS hydrogel lasted for 14 days, with more than 80% of Exos being smoothly released. This result ensured the achievement of optimal biological effects on the bone joints (Fig. 2I-J).

The results demonstrate the successful preparation of a cell-free hydrogel that mimics the extracellular matrix and is loaded with BMSCs-Exos.

### Enhanced chondrocyte repair and proliferation facilitated by GMOCS-exos hydrogel

**Fig. 3:**
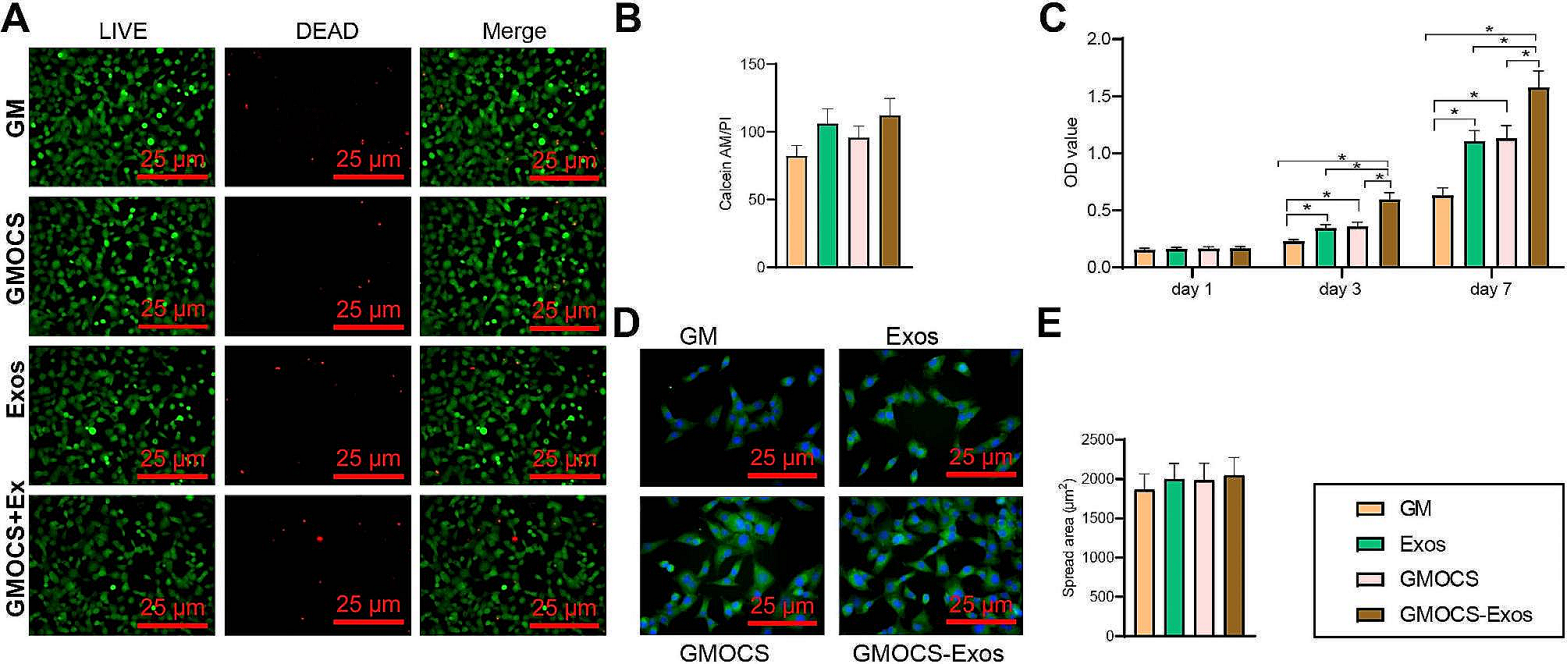
Biocompatibility Evaluation. *Note***(A)** Cell viability assay after 1 day of cell culture, green and red cells represent live and dead cells, respectively, scale bar = 200 μm; **(B)** Quantitative analysis of cell viability; **(C)** CCK-8 assay of cells cultured for 1, 3, and 7 days; **(D)** Cell adhesion of chondrocytes after 3 days of culture, green and blue represent Actin-Tracker Green-488 and Hoechst staining, respectively, scale bar = 100 μm; **(E)** Quantitative analysis of cell spreading area. Data are represented as mean ± standard deviation. Indicates *P* < 0.05, and cell experiments were repeated 3 times

To examine the biocompatibility of the GMOCS-Exos hydrogel, we conducted live/dead cell staining. As depicted in Fig. 3A, an amount of live cells (green) and minimal amounts of dead cells (red) were observed in all groups. The results of the CCK-8 analysis further demonstrate that chondrocyte proliferation increases over time in culture. The cell viability of the GMOCS and Exos groups is higher than that of the GM group on the 3rd and 7th day. However, the GMOCS-Exos group outperforms the GMOCS and Exos groups in enhancing chondrocyte proliferation. The results demonstrate that GMOCS-Exos can promote chondrocyte proliferation, as shown in Fig. 3B. Immunostaining of the cytoskeleton revealed that different groups of chondrocytes displayed an increased diffusion area, providing further evidence of the robust affinity and adhesive capacity of GMOCS-Exos towards chondrocytes (Fig. 3C-E).

**Fig. 4:**
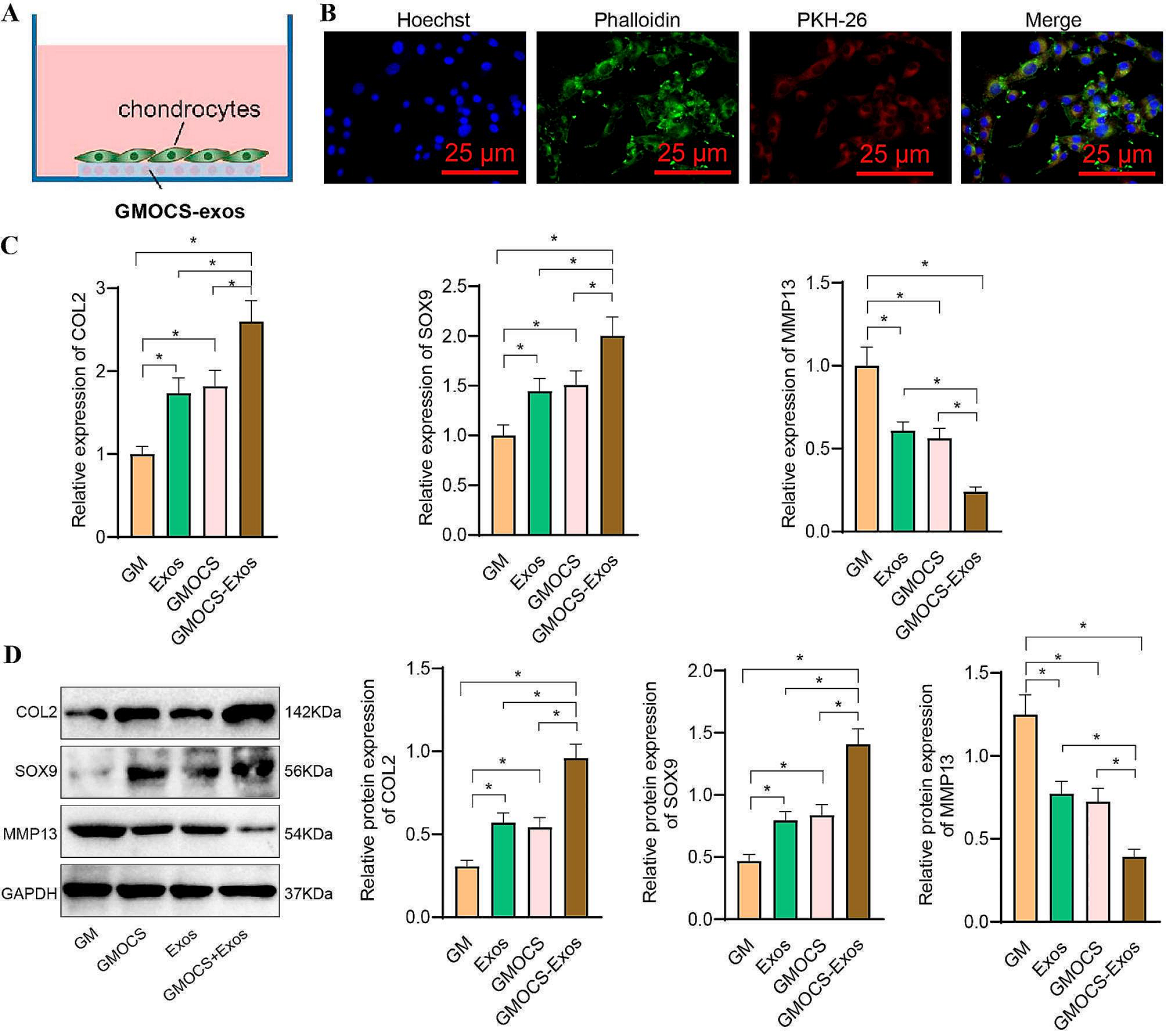
Promotion of GMOCS-Exos on Repair of Damaged Chondrocytes. *Note***(A)** Co-culture model of GMOCS-Exos/IL-1β pre-treated chondrocytes; **(B)** Immunofluorescence staining showing uptake of PKH26-labeled Exos by chondrocytes, scale bar = 50 μm; **(C)** mRNA levels of COL2, SOX9, and MMP13 detected by RT-qPCR; **(D)** Protein expression of COL2, SOX9, and MMP13 detected by Western blot. Data are represented as mean ± standard deviation. Indicates *P* < 0.05, and cell experiments were repeated 3 times

To assess the specific effect of GMOCS-Exos hydrogel on chondrogenesis, chondrocytes pretreated with IL-1β were cultured on the hydrogel for 7 days (Fig. 4A). Cellular skeleton staining demonstrates that chondrocytes absorb Exos on the GMOCS hydrogel after 24 h of co-culturing (Fig. 4B).

The results obtained from RT-qPCR analysis (Fig. 4C) demonstrated that the expression of SOX9 and COL2 was higher in the GMOCS group compared to the GM group. Conversely, the expression level of MMP13 was lower in the GMOCS group compared to the GM group. These findings indicate that OCS plays a role in chondrocyte repair. Furthermore, the expression levels of SOX9 and COL2 genes were higher in the Exos and GMOCS-Exos groups compared to the GM group. Notably, the GMOCS-Exos group exhibited the highest expression level. These findings suggest that Exos can alleviate IL-1β-induced damage in chondrocytes, and the combined treatment of GMOCS hydrogel and Exos can further enhance the repair of damaged chondrocytes.

The Western blot results demonstrated increased expression levels of SOX9 and COL2 proteins in the GMOCS-Exos group. Conversely, the expression level of MMP13 decreased, which aligns with RT-qPCR findings (Fig. 4D).

The results above indicate that GMOCS-Exos have the potential to promote the repair of that damaged chondrocytes.

### Therapeutic efficacy of GMOCS-Exos hydrogel in attenuating cartilage degeneration and bone loss in osteoarthritic rats

**Fig. 5:**
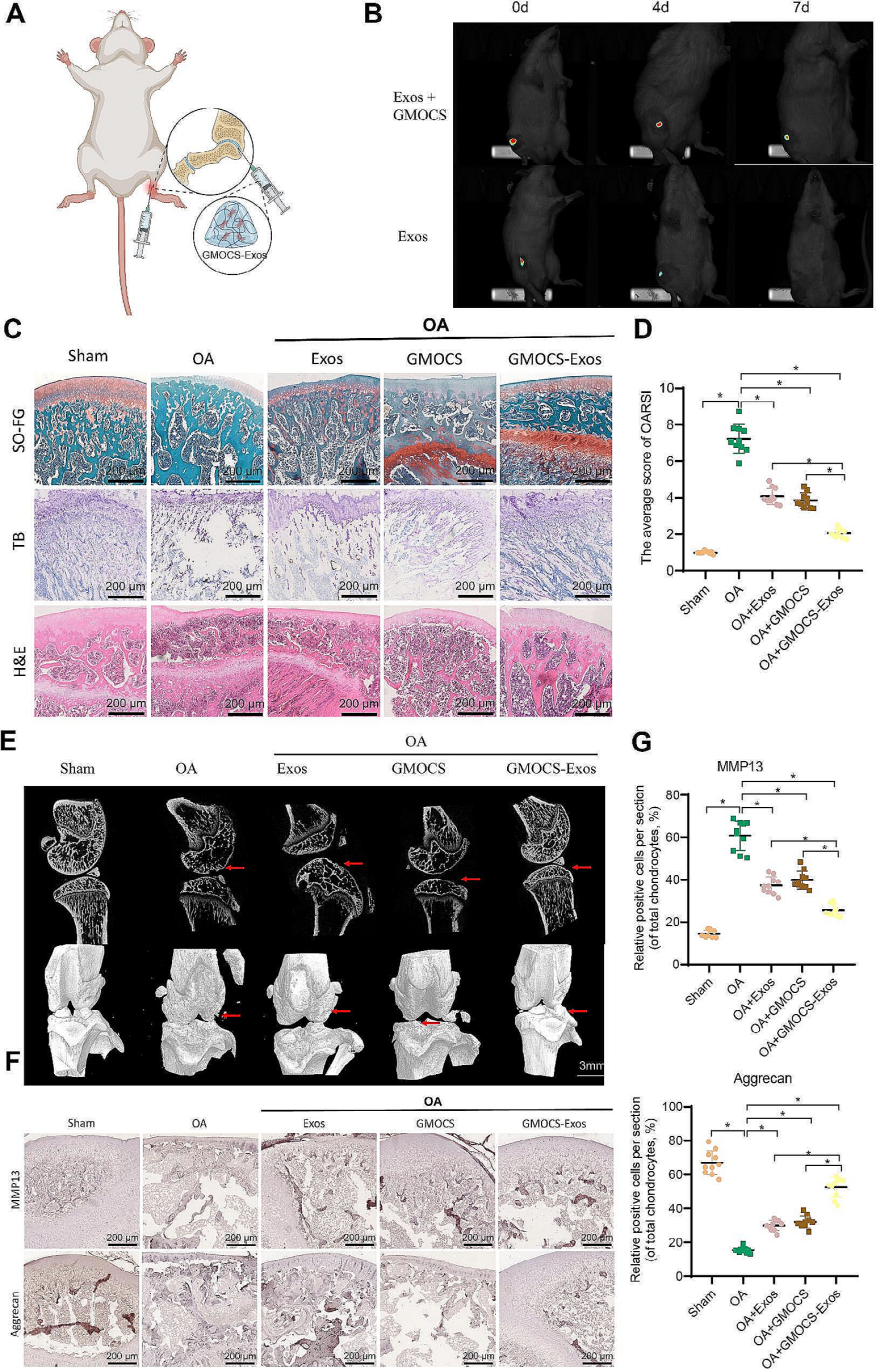
Effects of GMOCS-Exos on Knee Osteoarthritis in a Rat Model. *Note***(A)** Process of hydrogel implantation into the joint; **(B)** Retention ability of GMOCS-Exos; **(C)** Histological analysis of joint samples treated with different interventions using SO-FG, TB, and H&E staining; **(D)** OARIS scores of knee joint sections in each group; **(E)** Micro-CT analysis of knee joints in OA rats, with arrow indicating abnormal bone hypertrophy; **(F)** Immunohistochemical detection of MMP13 and Aggrecan expression levels, scale bar = 200 μm; **(G)** Quantitative analysis of immunohistochemical staining of MMP13 and Aggrecan. Data are represented as mean ± standard deviation. Indicates *P* < 0.05, *n* = 10

Motivated by the findings of in vitro investigations, we assessed the therapeutic efficacy of GMOCS-Exos hydrogel in osteoarthritis (OA) rats. Hemolysis testing demonstrates that hydrogels possess a high level of biocompatibility (Fig. S2A). Comparable to the control group, the OD value of the hydrogel group is below 0.2, suggesting that the GMOCS hydrogel does not induce hemolysis (Fig. S2B). Furthermore, the findings from the in vitro degradation experiment revealed a slow degradation rate of the hydrogel, which could be sustained for a minimum of 2 weeks. This finding ensures the prolonged release and therapeutic impact of Exos (Fig. S2C). The process of hydrogel implantation into bone joints is depicted in Fig. 5A. To ascertain the potential of GMOCS hydrogel in enhancing the in vivo retention of exosomes, we labeled the exosomes with PKH26 and observed the PKH26 fluorescence using an in vivo imaging system (IVIS). As illustrated in Fig. 5B, the fluorescence intensity in the Exos group decreased on day 4 and reached an extremely low level by day 7. The fluorescence decay rate of the GMOCS-Exos group exhibits a lower value. GMOCS-Exos has been demonstrated to increase the duration of Exos retention in the body and improve their stability, resulting in long-term effects of GMOCS-Exos.

Following the successful establishment of an osteoarthritis (OA) rat model, we conducted Safranin O/Fast Green, TB, and H&E staining on the sections of the rat knee joint (Fig. 5C). The findings suggest that rats with OA exhibit clear indications of cartilage degeneration, such as matrix degradation and erosion. In contrast, the thickness and density of the cartilage matrix increased in the Exos group, GMOCS group, and GMOCS-Exos group, thereby confirming their efficacy in delaying cartilage degeneration. The cartilage repair effect of the GMOCS-Exos group is more pronounced, attributed to the hydrogel’s long-term storage effect. Furthermore, the OARSI score in rats, conducted by the International Osteoarthritis Research Society, confirmed the protective effect of GMOCS-Exos on osteoarthritis (Fig. 5D).

Micro-CT imaging was employed to evaluate the alterations in the subchondral bone. Compared to the Sham group, the knee joints of OA rats exhibited a considerable increase in osteophyte formation. The Exos and GMOCS groups demonstrated a decrease in osteophyte formation compared to the OA group. Furthermore, the reduction in osteophyte formation was more pronounced following GMOCS-Exos treatment (Fig. 5E), suggesting the protective effect of GMOCS-Exos on OA. Furthermore, the Immunohistochemistry results demonstrated a lower expression level of Aggrecan in the OA group compared to the Sham group and a higher expression level of MMP13. The expression of Aggrecan was increased in the GMOCS-Exos group, whereas the expression of MMP13 was decreased (Fig. 5F-G).

The results above demonstrate the beneficial impact of GMOCS-Exos on cartilage protection in the body. Specifically, GMOCS-Exos alleviates cartilage lesions and subchondral bone loss in rats with osteoarthritis (OA).

### Inhibition of NET formation by GMOCS-Exos through activation of the Nrf2 pathway

**Fig. 6:**
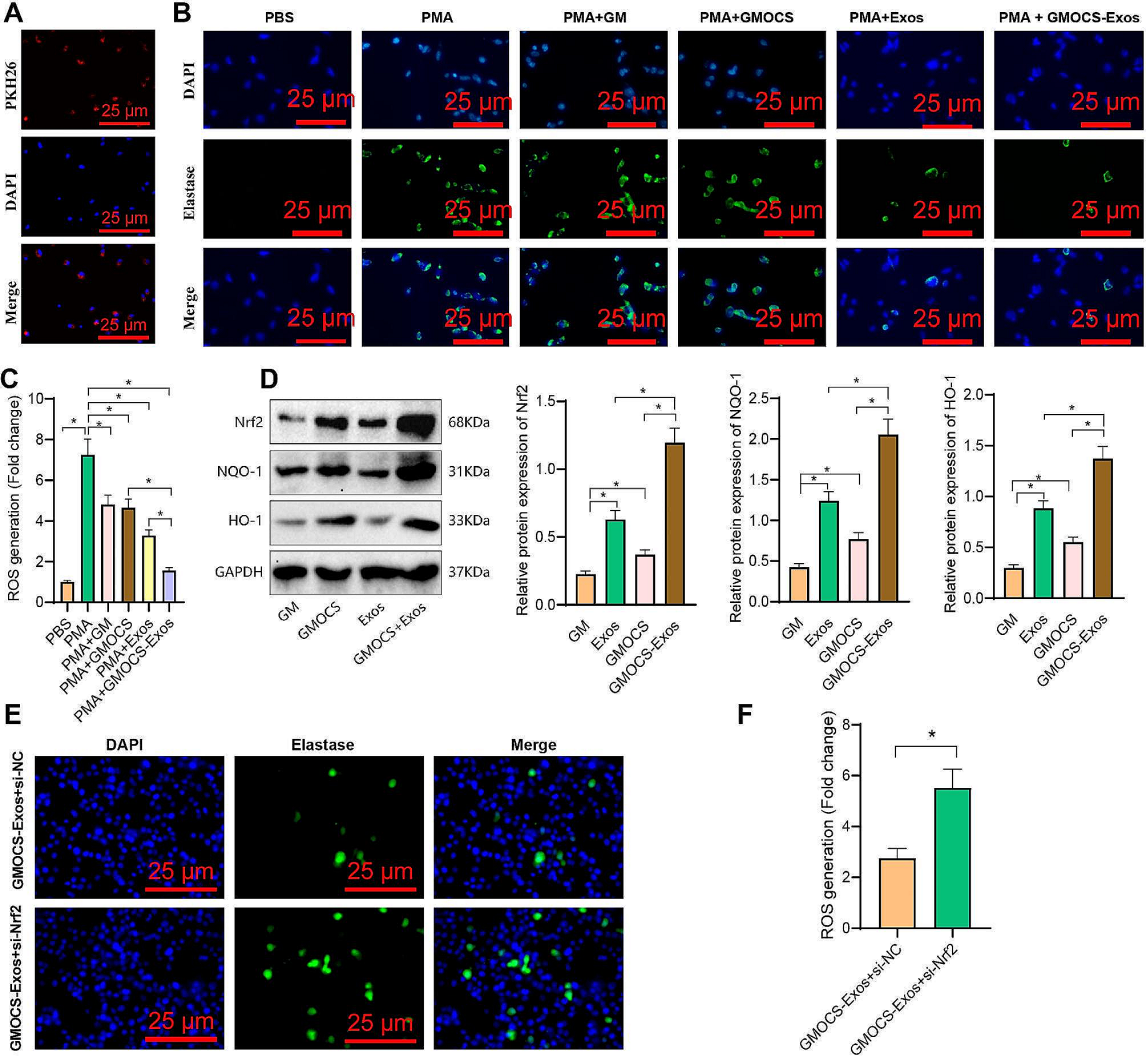
Regulation of Neutrophils by GMOCS-Exos via the Nrf2 Pathway. *Note***(A)** Fluorescence microscopy observation of GMOCS-exos uptake by neutrophils, scale bar = 10 μm; **(B)** Immunofluorescence detection of elastase expression, scale bar = 50 μm; **(C)** Quantification of ROS release by flow cytometry; **(D)** Protein expression levels of Nrf2, HO-1, and NQO-1 detected by Western blot; **(E)** Immunofluorescence detection of NETs formation in Nrf2-silenced cells, scale bar = 50 μm; **(F)** Effects of Nrf2 silencing on ROS release by flow cytometry. Data are represented as mean ± standard deviation. Indicates *P* < 0.05, and cell experiments were repeated 3 times

Previous studies have demonstrated the presence of neutrophil elastase-mediated degradation of cartilage in NETs [51]. To assess the impact of GMOCS-exos on NET formation, we obtained neutrophils from the synovial fluid of patients with osteoarthritis (OA) and treated them with PMA. Subsequently, the neutrophils were co-cultured with GMOCS-exos. Figure 6A demonstrates that neutrophils could engulf Exos loaded on GMOCS hydrogel. The confocal microscopy results (Fig. 6B) demonstrate that after a 2-hour incubation of neutrophils with PMA, the levels of NETs markers in the GMOCS-Exos and Exos groups are lower compared to the GM group. No statistical difference was found between the GMOCS and GM groups, suggesting that GMOCS had no impact on NETs. In contrast, GMOCS-Exos were found to disrupt the formation of NETs.

Furthermore, the formation of NETs is associated with the generation of reactive oxygen species (ROS) [52]. Reactive oxygen species (ROS) production was observed in neutrophils co-cultured with GMOCS-Exos following PMA treatment. As depicted in Fig. 6C, applying PMA augmented the release of reactive oxygen species (ROS), whereas the GMOCS-Exos group demonstrated a notable reduction in ROS release.

Research has revealed that Nrf2 serves as a transcriptional regulatory factor implicated in various biological processes, including the response to oxidative stress, antioxidant defense, cellular detoxification, drug transport, and cellular protection through the activation of specific genes. The involvement of Nrf2 has been documented in osteoarthritis and neutrophil aging [53].

Our findings indicate that Nrf2, HO-1, and NQO-1 expression was upregulated in both the GMOCS-Exos and Exos groups, compared to the GM group. However, no statistical difference was observed between the GMOCS and GM groups (Fig. 6D). Furthermore, the decrease in NET formation and release of reactive oxygen species (ROS) observed in the GMOCS-Exos group was negated by introducing sh-Nrf2 (Fig. 6E-F).

The results above indicate that GMOCS-Exos can disrupt NETs by activating the Nrf2 pathway.

### Promotion of cartilage regeneration by GMOCS-exos through inhibition of NETs and stimulation of chondrocyte proliferation, migration, and viability

**Fig. 7:**
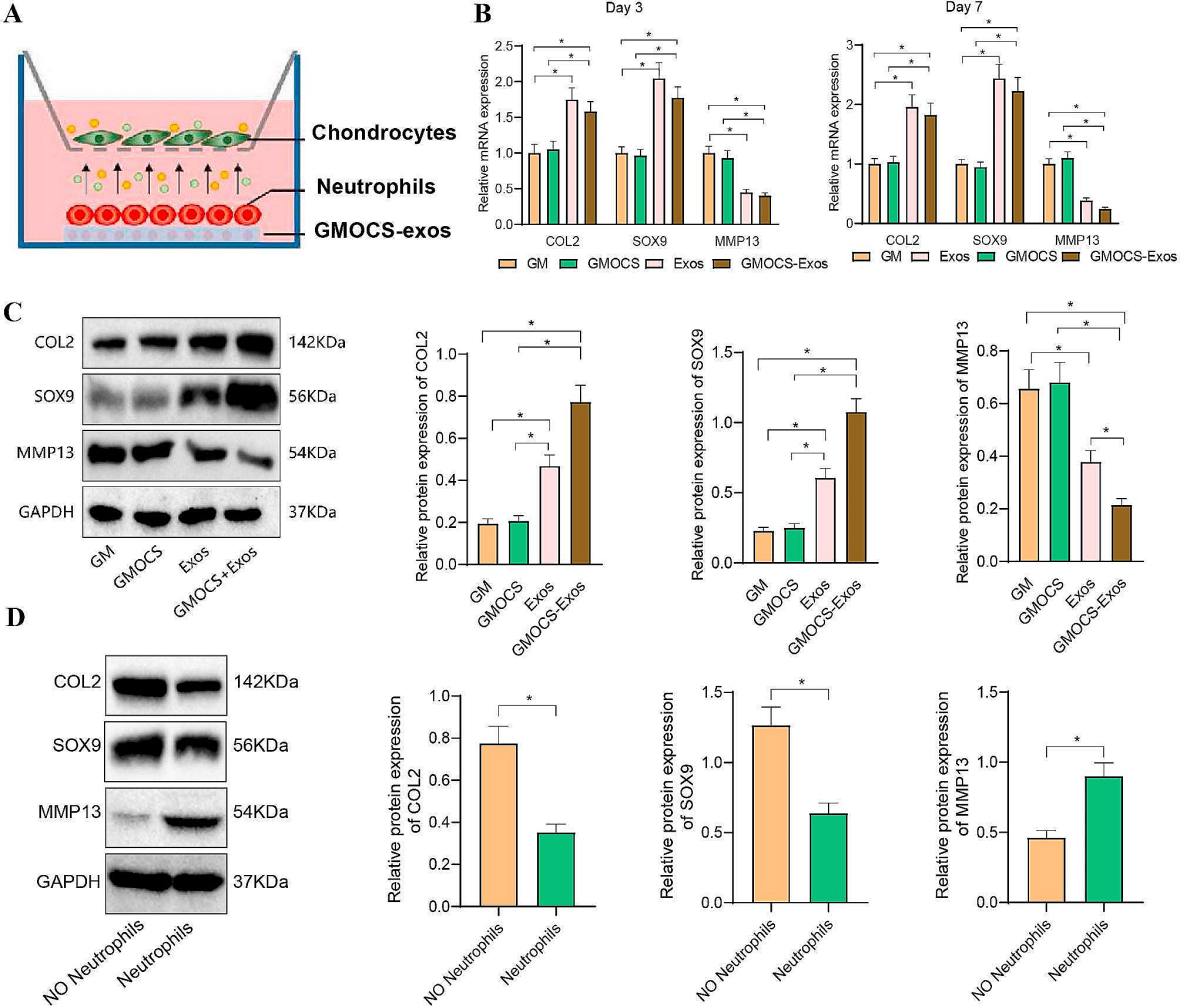
Protective Effect of Neutrophils on Cartilage ECM Degradation by GMOCS-Exos. *Note***(A)** Co-culture model of GMOCS-Exos/neutrophils/damaged chondrocytes; **(B)** mRNA levels of COL2, SOX9, and MMP13 detected by RT-qPCR; **(C)** Protein expression levels of COL2, SOX9, and MMP13 detected by Western blot; **(D)** Western blot analysis of GMOCS-Exos/chondrocytes co-cultured with or without neutrophils. Data are represented as mean ± standard deviation. Indicates *P* < 0.05, and cell experiments were repeated 3 times

The above description illustrates the association between GMOCS-exos and neutrophils. Subsequently, we examined the impact of this interaction on chondrocytes. We have established a GMOCS-exos/neutrophil/chondrocyte co-culture system (Fig. 7A). Chondrocytes were pre-treated with IL-1β before the experiment and co-cultured with PMA-treated neutrophils for 3 and 7 days to assess chondrogenesis. The RT-qPCR results (Fig. 7B) demonstrated that both the GMOCS-Exos group and the Exos group increased the levels of COL2 and SOX9 in damaged chondrocytes while decreasing the expression of MMP13. No difference was observed between the Exos and GMOCS-Exos groups on the third day.

However, on the 7th day, the therapeutic effect of the GMOCS-Exo group was superior to that of the Exos group. This difference could be attributed to the GMOCS-Exo group’s sustained release effect and ability to inhibit NETs formation. The Western blot and RT-qPCR results were consistent on the 7th day of co-cultivation. They indicated that the GMOCS-Exos treated group showed the highest expression of COL2 protein, while the expression of MMP13 protein was the lowest (Fig. 7C).

Furthermore, Western blot analysis was conducted after 7 days of co-culturing neutrophils with GMOCS-Exos/chondrocytes, with or without PMA treatment, to further validate the impact of neutrophils. The results imply that the chondrogenic potential of GMOCS-Exos experienced an increase in the absence of neutrophils (Fig. 7D).

The results of the research above indicate that the sustained release of Exos in hydrogel could enhance the synthesis of extracellular matrix (ECM) in cartilage cells by inhibiting the formation of neutrophil extracellular traps (NETs).

**Fig. 8:**
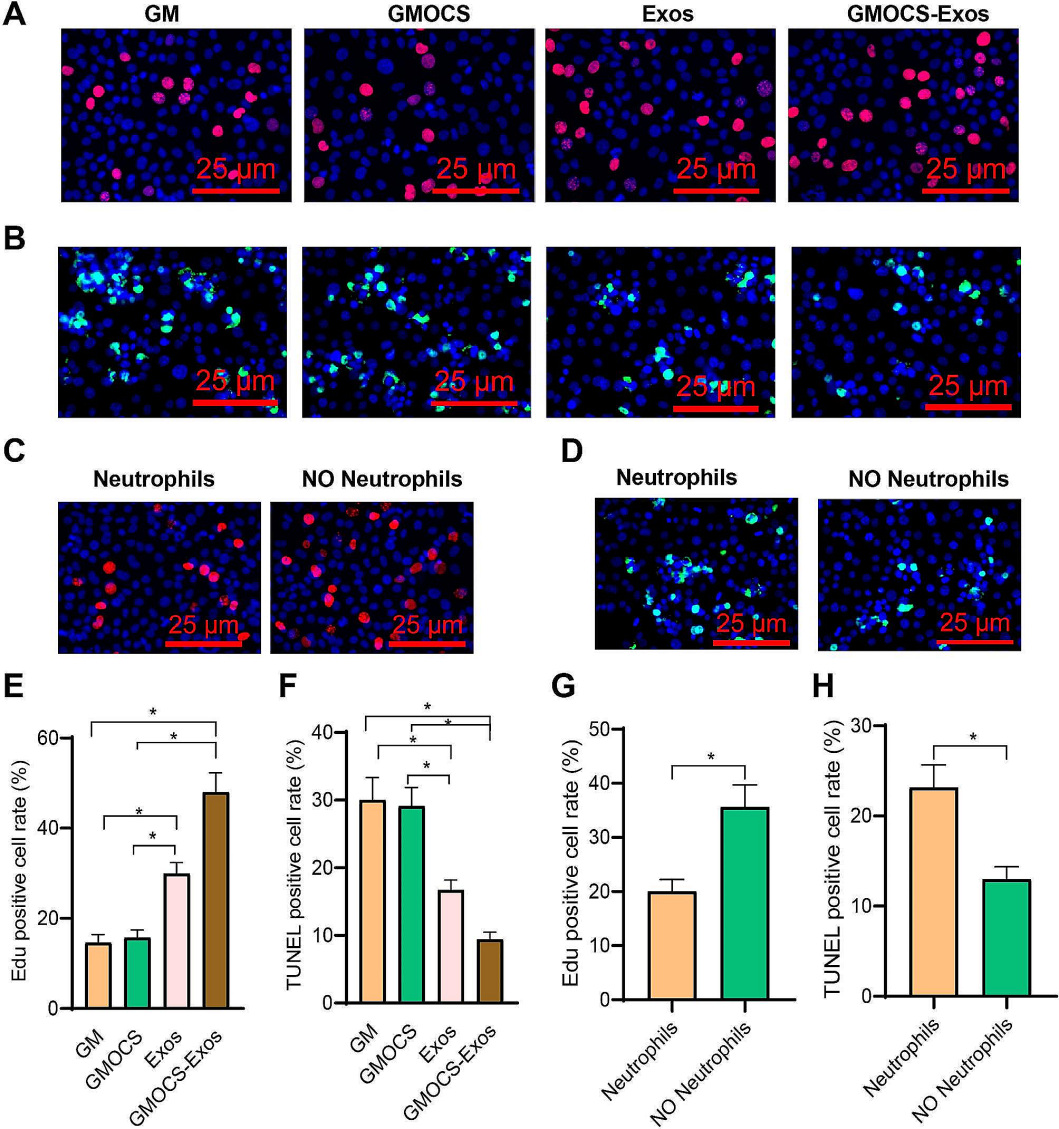
Effects of Neutrophils on Proliferation and Apoptosis of Chondrocytes by GMOCS-Exos. *Note***(A)** EdU staining to measure the proliferation rate of chondrocytes, scale bar = 200 μm; **(B)** Immunofluorescence staining to measure the apoptosis rate of chondrocytes, scale bar = 200 μm; **(C)** Proliferation rate of chondrocytes co-cultured with GMOCS-Exos with or without neutrophils, scale bar = 200 μm; **(D)** Apoptosis rate of chondrocytes co-cultured with GMOCS-Exos with or without neutrophils, scale bar = 200 μm; **(E**&**G)** Analysis of results from EdU staining and flow cytometry; **(F**&**H)** Analysis of TUNEL-positive cell rate in each group. Data are represented as mean ± standard deviation. Indicates *P* < 0.05, and cell experiments were repeated 3 times

The results of EdU immunofluorescence demonstrated an enhanced proliferation rate of chondrocytes in both the GMOCS-Exos and the Exos groups (Fig. 8A and E), displaying statistical differences. The results of the TUNEL apoptosis detection indicated a reduction in the apoptotic rate of chondrocytes in both the GMOCS-Exos and the Exos groups (Fig. 8B and F).

Additionally, we conducted EdU and TUNEL analysis (Fig. 8C, G, D and H) after co-culturing with GMOCS-Exos/chondrocytes for 7 days, with or without PMA treatment of neutrophils. The study findings demonstrated an increase in the chondrogenic effect of GMOCS-Exos when neutrophils were absent. The results reveal that GMOCS-Exos stimulates the proliferation of chondrocytes and prevents chondrocyte apoptosis by inhibiting the formation of NETs.

**Fig. 9:**
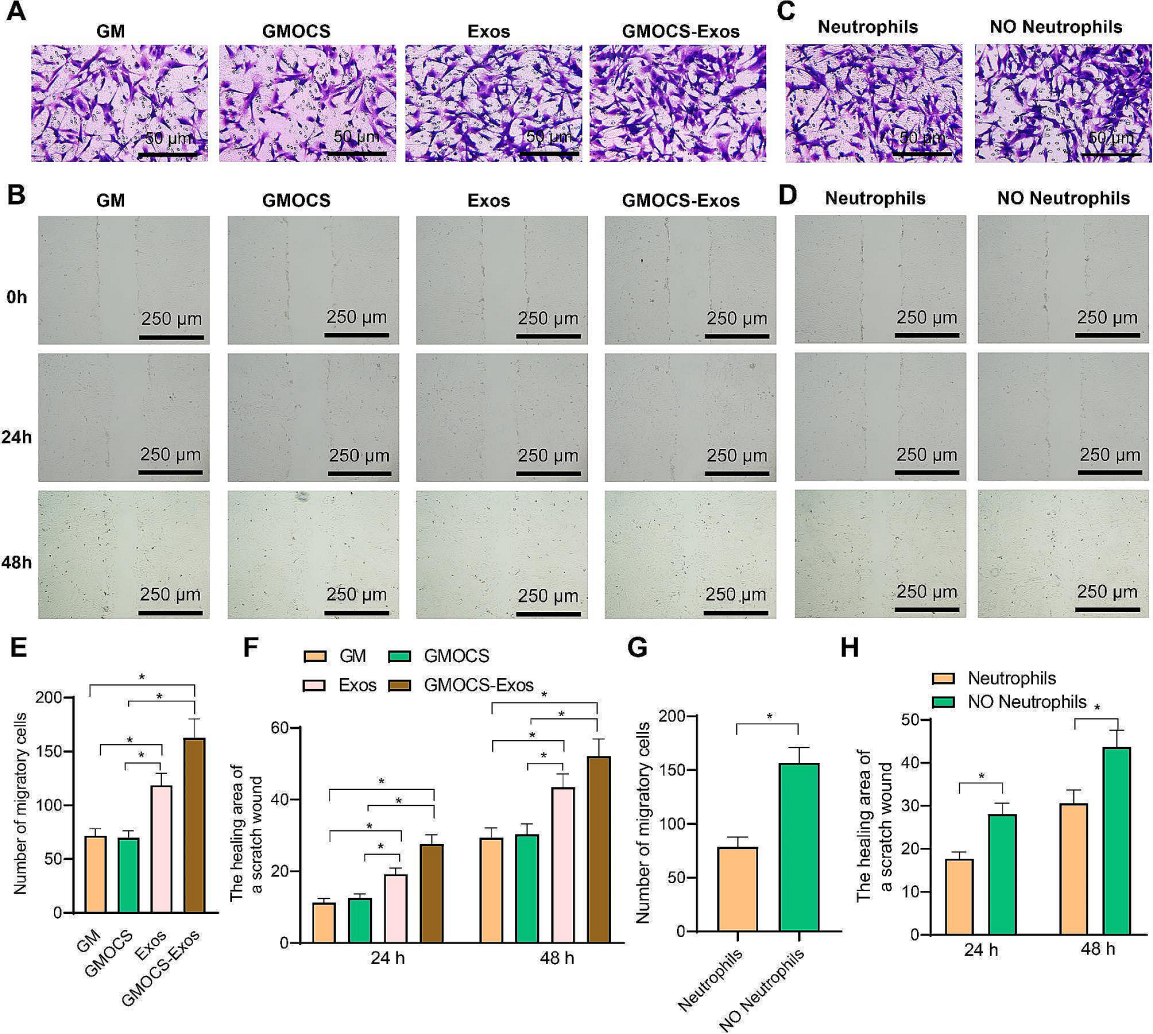
Influence of GMOCS-exos on the migration of chondrocytes through neutrophils. *Note***(A)** Transwell assay to observe the migration of chondrocytes, scale bar = 200 μm; **(B)** Microscope images of the scratch area healing of chondrocytes at 0 h, 24 h, and 48 h, scale bar = 200 μm; **(C)** Migration of chondrocytes after co-culture with GMOCS-Exos/chondrocytes with or without neutrophils, scale bar = 200 μm; **(D)** Scratch area healing of chondrocytes after co-culture with GMOCS-Exos/chondrocytes with or without neutrophils, scale bar = 200 μm; **(E**&**G)** Analysis of Transwell assay results; **(F**&**H)** Analysis of scratch assay results. Data presented as mean ± standard deviation. * represents *P* < 0.05. The cell experiments were repeated 3 times

To assess the migratory capacity of chondrocyte groups, we employed the Transwell and cell scratch assays for wound healing. The Transwell assay results showed increased chondrocyte migration in both the GMOCS-Exos and the Exos groups, as depicted in Fig. 9A and E, respectively. The scratch assay of cells consistently yielded results demonstrating that treatment with GMOCS-Exos and Exos improved the healing ability of chondrocytes co-cultured with neutrophils (Fig. 9B and F).

In addition, we conducted Transwell assays (Fig. 9C and G) and cell scratch assays (Fig. 9D and H) after co-culturing with GMOCS-Exos/chondrocytes for 7 days, regardless of the presence of neutrophils treated with PMA. It was discovered that in the absence of neutrophils, the migratory capacity of GMOCS-Exos in chondrocytes increased significantly. This result suggests that GMOCS-Exos enhances chondrocyte migration through inhibition of NETs formation.

The research above results suggests that the sustained release of Exos in GMOCS hydrogel can potentially promote cartilage regeneration by suppressing the formation of NETs.

### GMOCS-exos attenuates osteoarthritis progression in rats through activation of the Nrf2 pathway

**Fig. 10:**
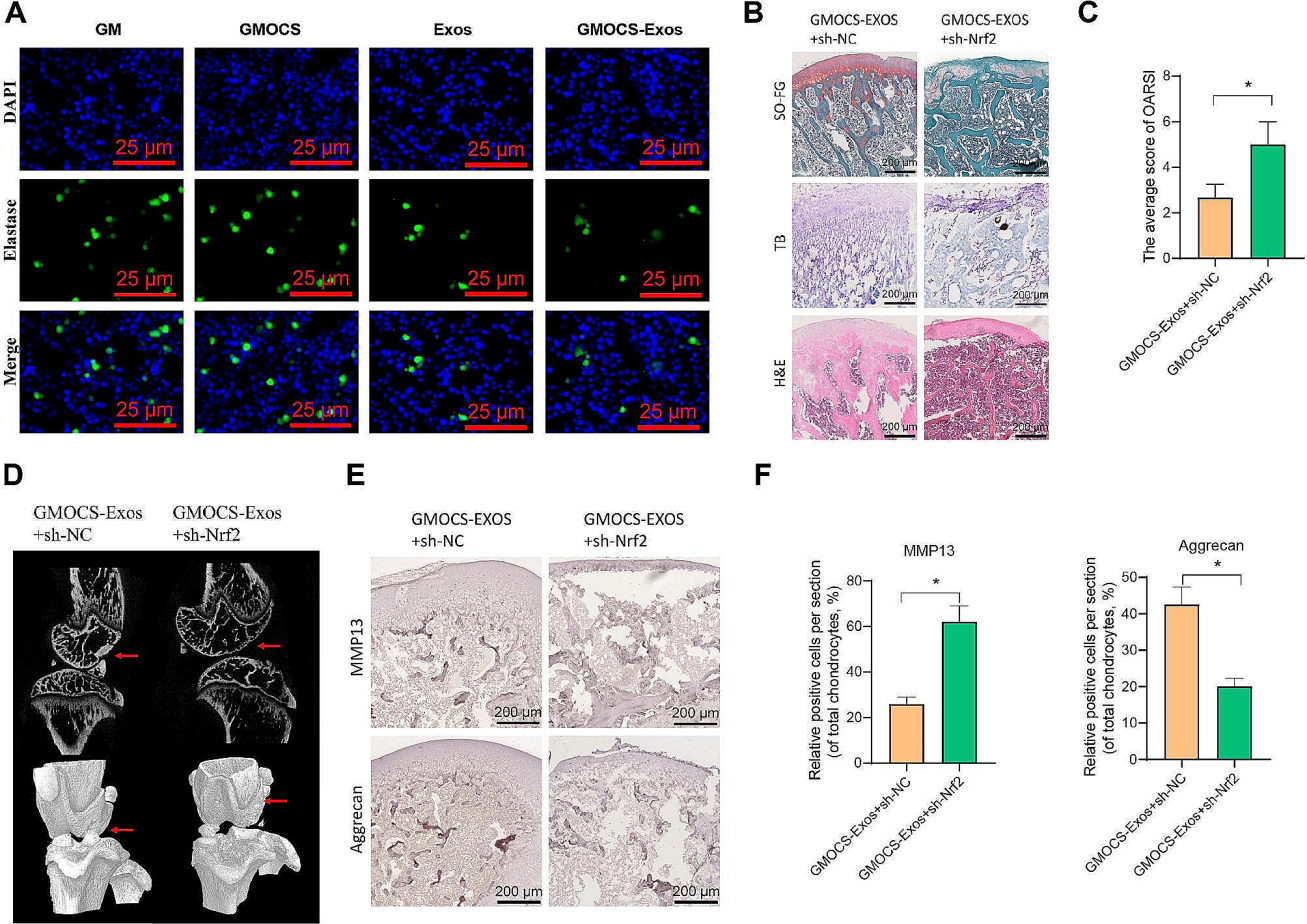
Influence of GMOCS-exos on knee osteoarthritis in an OA rat model through Nrf2. *Note***(A)** Immunofluorescence detection of elastase expression, scale bar = 50 μm; **(B)** Histological analysis of joint samples from different treatments using SO-FG, TB, and H&E staining images; **(C)** OARIS scores of knee joint sections in each group; **(D)** Micro-CT analysis of OA rat knee joints, arrows indicate abnormal bone hypertrophy; **(E)** Immunohistochemical detection of MMP13 and Aggrecan expression levels, scale bar = 200 μm; **(F)** Quantitative analysis of MMP13 and Aggrecan immunohistochemistry. Data presented as mean ± standard deviation. * represents *P* < 0.05. *n* = 10

To investigate the impact of GMOCS-Exos on the formation of NETs in rats with osteoarthritis (OA), we obtained neutrophils from the synovial fluid of OA rats. The neutrophils were then treated with PMA and co-cultured with GMOCS-Exos. As depicted in Fig. 10A, the expression of NETs markers was lower in the GMOCS-Exos group than in the GM and GMOCS groups. No difference was observed between the GMOCS and GM groups, suggesting that the independent hydrogel implantation does not exert a regulatory effect on NETs.

Moreover, the absence of Nrf2 in OA rats was confirmed through Safranin O/Fast Green, TB, and H&E staining, indicating that GMOCS-Exos no longer protected the articular cartilage of the rat’s knee (Fig. 10B). The OARSI score further validated the diminished chondroprotective capability of GMOCS-Exos, as evident in Fig. 10C. Micro-CT images of rat knee joints demonstrated that the inhibitory effect of GMOCS-Exos on osteophyte formation was blocked when Nrf2 was silenced (Fig. 10D). Furthermore, immunohistochemical results demonstrated that the suppression of Nrf2 prevented the notable upregulation of Aggrecan expression and downregulation of MMP13 expression induced by GMOCS-Exos (Fig. 10E-F).

The results above indicate that GMOCS-Exos hinders the progression of rat osteoarthritis by activating the Nrf2 pathway.

### TGFB1 mediates the therapeutic effects of GMOCS-exos through activation of the Nrf2 signaling pathway in neutrophils

**Fig. 11:**
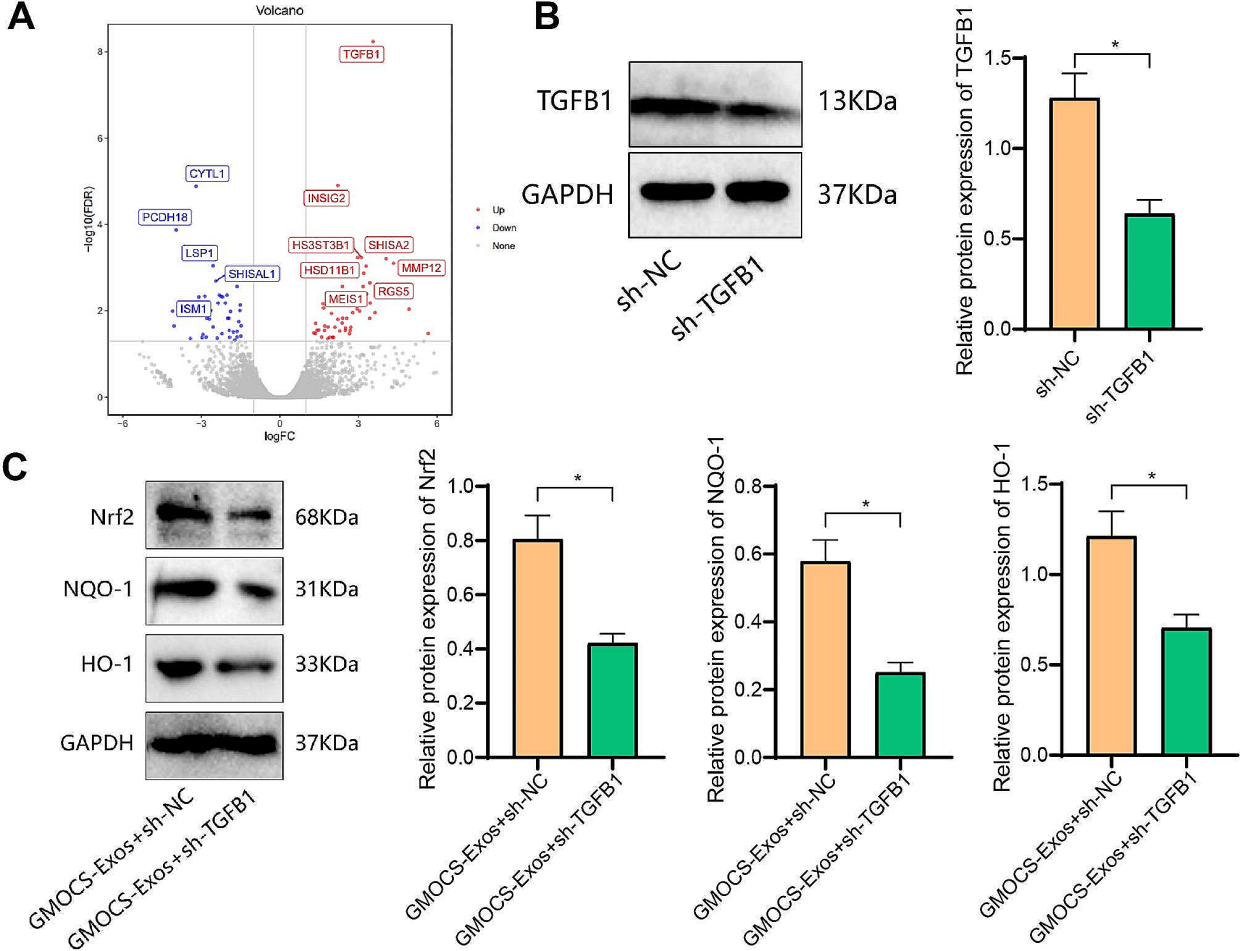
TGFB1 is a key gene regulating the Nrf2 signaling pathway in GMOCS-Exos-treated chondrocytes. *Note***(A)** Volcano plot of the top 10 differentially expressed genes in chondrocytes treated with GMOCS-Exos; **(B)** Western blot detection of TGFB1 protein expression in neutrophils; **(C)** Western blot detection of Nrf2, HO-1, and NQO-1 protein expression in neutrophils. Data presented as mean ± standard deviation. * represents *P* < 0.05. The cell experiments were repeated 3 times

Both in vitro and in vivo studies have demonstrated the therapeutic effects of GMOCS-Exos. However, additional research is required to investigate the fundamental molecules involved in the regulatory process. Total RNA was isolated from chondrocytes co-cultured with GMOCS-Exos, and subsequent RNA-seq analysis identified TGFB1 as the most upregulated gene in these chondrocytes (Fig. 11A).

To further validate the role of TGFB1, it was silenced in neutrophils (Fig. 11B). We utilized GMOCS-Exos to process both normal neutrophils and TGFB1-silenced neutrophils, followed by Western blot analysis. The results indicate that sh-TGFB1 strongly suppresses the Nrf2 signaling pathway in neutrophils following GMOCS-Exos treatment, thereby diminishing the anti-inflammatory effect of Exos (Fig. 11C).

It indicates that TGFB1 plays a critical role in mediating the therapeutic effects of GMOCS-Exos by activating the Nrf2 signaling pathway in neutrophils.

## Suppression of TGFB1 negates the chondroprotective effect of GMOCS-exos in a rat model of osteoarthritis

**Fig. 12:**
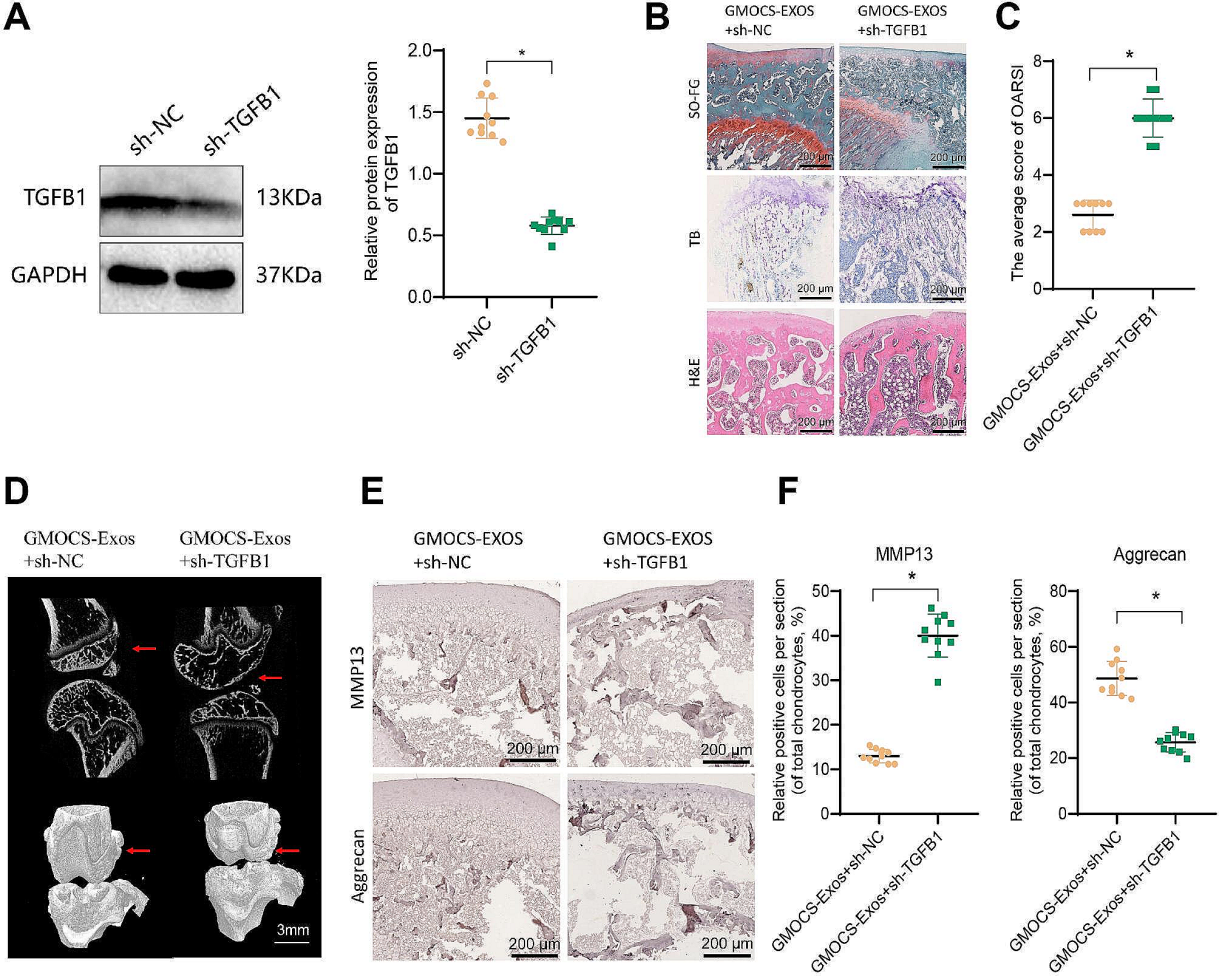
Influence of TGFB1 on knee osteoarthritis. *Note***(A)** Western blot detection of TGFB1 protein expression; **(B)** Histological analysis of joint samples from different treatments using SO-FG, TB, and H&E staining images; **(C)** OARIS scores of knee joint sections in each group; **(D)** Micro-CT analysis of OA rat knee joints, arrows indicate abnormal bone hypertrophy; **(E)** Immunohistochemical detection of MMP13 and Aggrecan expression levels, scale bar = 200 μm; **(F)** Quantitative analysis of MMP13 and Aggrecan immunohistochemistry. Data presented as mean ± standard deviation. * represents *P* < 0.05. *n* = 10

To further study the involvement of TGFB1 in the protection of intra-articular cartilage by GMOCS-exos in a rat model of osteoarthritis, we suppressed TGFB1 expression using sh-TGFB1 in rats (Fig. 12A). Safranin O and Fast Green are commonly utilized to stain samples during sectioning. The results indicate that sh-TGFB1 rats exhibited cartilage damage characterized by matrix degradation and erosion. These rats also had higher OARSI scores than sh-NC rats (Fig. 12B-C). Micro-CT imaging of the rat knee joint showed a notable augmentation in osteophyte formation when sh-TGFB1 was present (Fig. 12D).

Furthermore, immunohistochemistry results demonstrated that the inhibition of TGFB1 prevented the reduction in MMP13 expression and markedly enhanced Aggrecan expression induced by GMOCS-Exos (Fig. 12E-F). These findings indicate that depleting TGFB1 in the body hinders the chondroprotective effect of GMOCS-exos.

## GMOCS-exos enhances aggrecan expression and suppresses MMP13 in human osteoarthritic cartilage

**Fig. 13:**
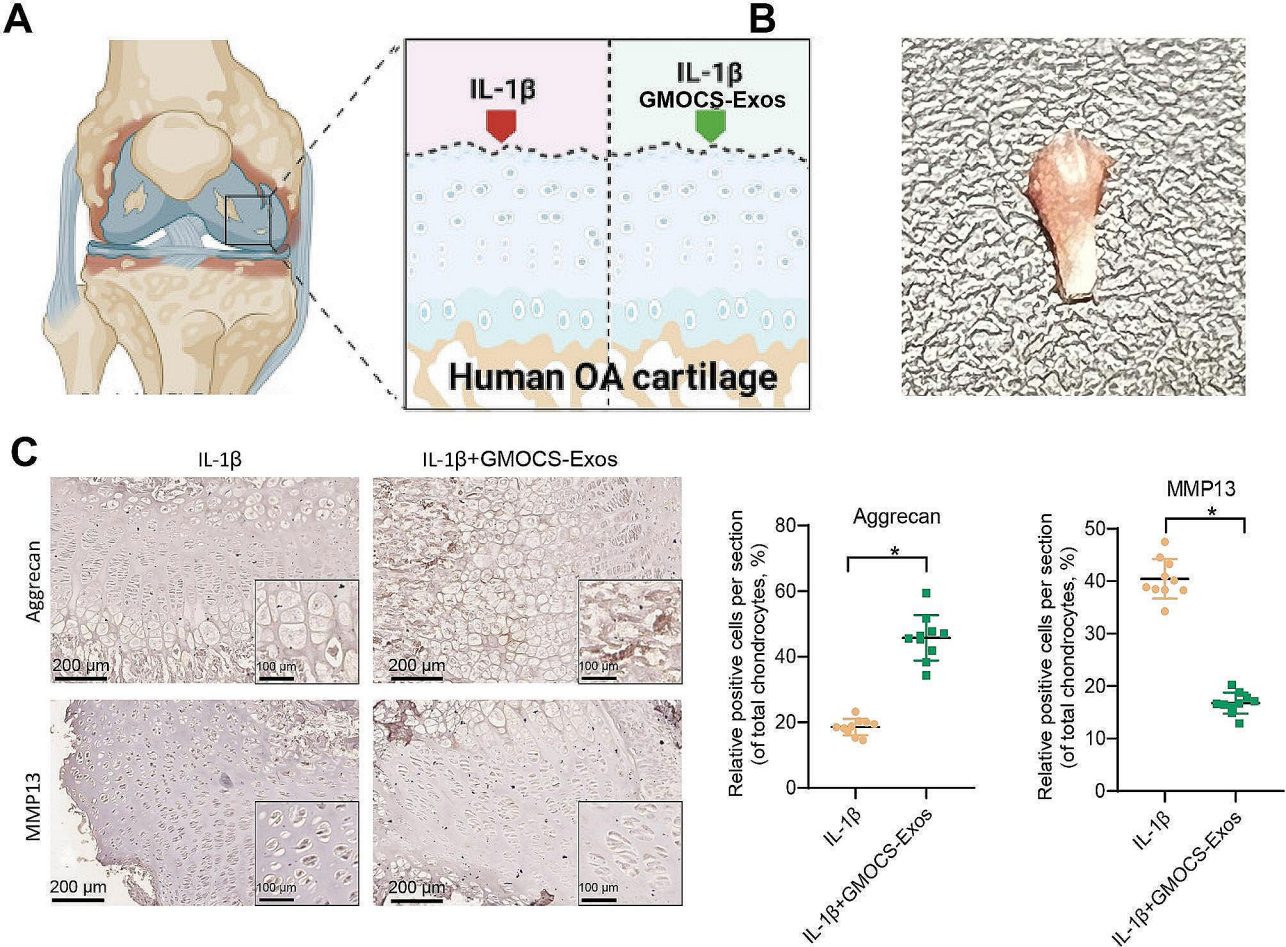
The therapeutic effect of GMOCS-Exos on human OA cartilage explants. *Note***(A)** Schematic diagram of the harvesting and processing of human OA cartilage explants; **(B)** Representative images of harvested human OA cartilage explants; **(C)** Immunohistochemical detection of Aggrecan and MMP13 expression levels in IL-1β-maintained explants with or without GMOCS-Exos, scale bar = 200 μm. Data presented as mean ± standard deviation. *n* = 3 per group. * represents *P* < 0.05

To explore the clinical applications of GMOCS-Exos, we divided the cartilage grafts from a single patient who underwent TKA into two groups. One group was treated with GMOCS-Exos for 72 h, while the other group did not receive any treatment. To preserve the osteoarthritis (OA) phenotype, chondrocyte autografts were cultured in a medium supplemented with human IL-1β (10 ng/mL) (Fig. 13A-B).

Immunohistochemical analysis of the immune system revealed increased Aggrecan expression and suppressed MMP13 expression in human OA cartilage after treatment with GMOCS-Exos (Fig. 13C). It suggests that GMOCS-Exos has potential in the treatment of human OA.

## Discussion

**Fig. 14:**
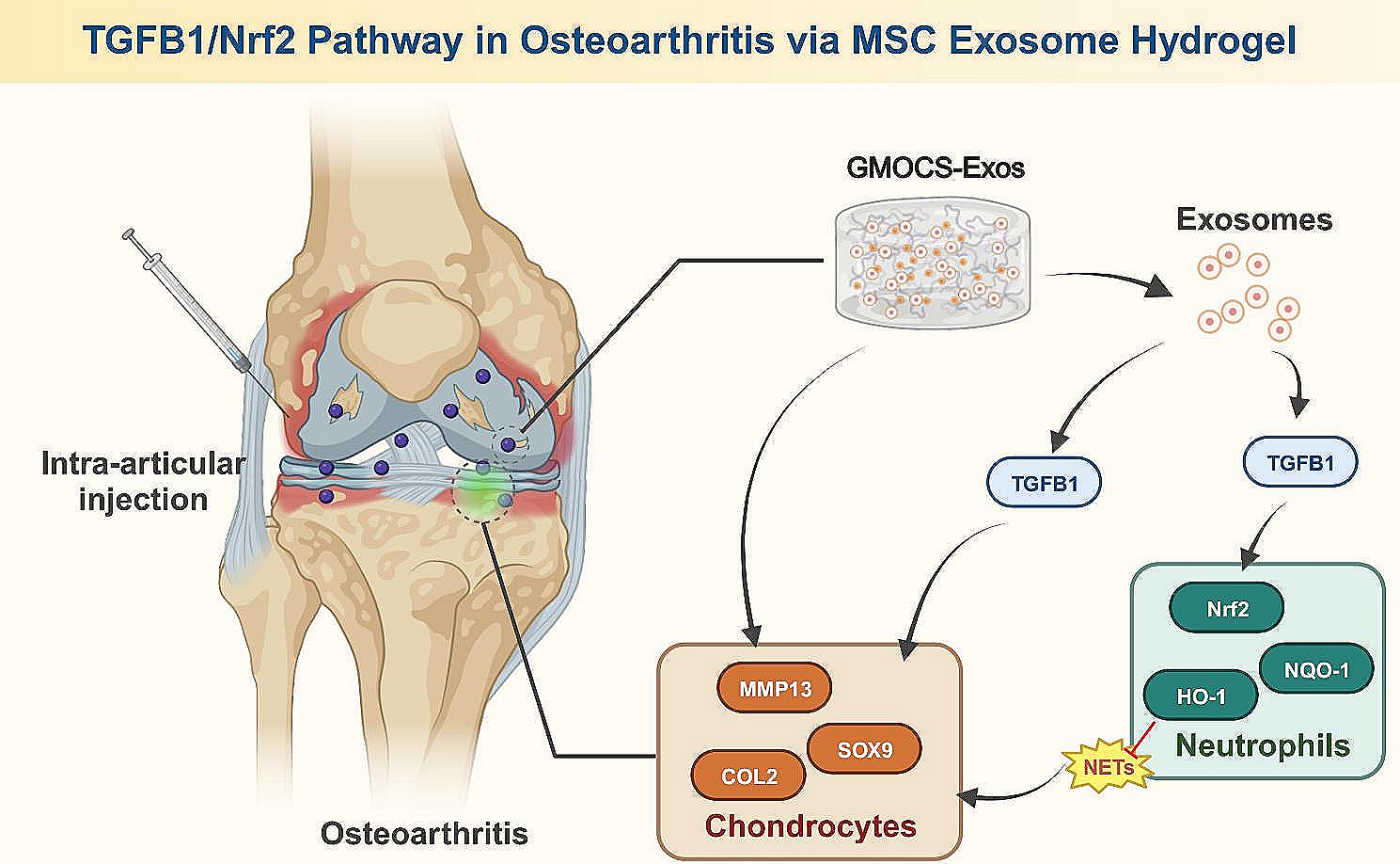
Mechanism of the TGFB1/Nrf2 signaling pathway mediated by extracellular matrix-mimicking hydrogel loaded with bone marrow mesenchymal stem cell exosomes in osteoarthritis

This study discovered a novel mechanism by which the extracellular matrix mimics a hyaluronic acid gel loaded with exosomes derived from mesenchymal stem cells. This mechanism enhances the activation of the Nrf2 signaling pathway, which TGFB1 mediates, and simultaneously disrupts neutrophil extracellular traps (NETs). As a result, it effectively alleviates cartilage degeneration in osteoarthritis, as illustrated in Fig. 14. This study offers a novel perspective for comprehending osteoarthritis’s pathophysiological mechanisms and treatment. Our research objective has been successfully achieved based on the experimental data, offering new and promising strategies for treating osteoarthritis.

There are evident similarities and differences when comparing this study to previous studies. Previous studies predominantly relied on conventional biotechnological techniques to assess cell infiltration and activation of signal pathways. Nonetheless, our research has employed cutting-edge bioinformatics tools like ESTIMATE and MCPcounter, facilitating a more accurate evaluation of neutrophil infiltration [54]. Furthermore, our transcriptome sequencing analysis has unveiled a strong correlation between the TGFB1 and Nrf2 signaling pathways, offering novel research insights in this area [55].

The TGFB1/Nrf2 signaling pathway plays a role in several diseases, particularly those associated with oxidative stress and inflammation [23, 56, 57]. Previous studies have demonstrated that this signaling pathway exhibits antioxidative and anti-inflammatory effects in various other diseases [58]. Our research further confirms the important role of neutrophil immune infiltration in osteoarthritis.

Neutrophils, a vital constituent of the innate immune system, play a pivotal role in the inflammatory response of numerous diseases [59, 60]. In the case of osteoarthritis, the presence of neutrophils infiltrating the immune system is strongly linked to the progress and seriousness of the condition [61]. Moreover, neutrophils are also acknowledged as key factors in the progression of other diseases, including rheumatoid arthritis and inflammatory bowel disease [62].

In recent years, due to their substantial potential, there has been a growing research interest in mesenchymal stem cell-derived extracellular vesicles, specifically in tissue repair and regenerative medicine [63]. Our research has discovered that extracellular vesicles derived from mesenchymal stem cells found in bone marrow, which contain TGFB1, possess the potential to regulate the progression of osteoarthritis. In contrast to other diseases, such as heart disease and diabetes, the extracellular vesicles derived from bone marrow mesenchymal stem cells have also exhibited substantial therapeutic effects [64].

This discovery emphasizes the pivotal role of neutrophils in osteoarthritis development, offering a theoretical foundation for advancing more focused treatment strategies in clinical practice. Therapeutic effects of extracellular vesicles derived from mesenchymal stem cells have been observed in osteoarthritis, indicating that exploiting this mechanism could become a promising approach for future clinical treatment. The study sample size may impose limitations on the generalizability of the findings. Insufficient sample size or biased selection of samples may render the conclusions inapplicable to all osteoarthritis patients.

Similar to all research, potential biases or errors in experimental design could exist. These factors could impact the study’s results, necessitating further verification and adjustment. While studies have established a connection between exosomes derived from mesenchymal stem cells and the Nrf2 signaling pathway, further investigation is required to determine the specific molecular and cellular mechanisms involved.

Future studies could expand upon our research findings and provide more insight into the specific mechanism of the TGFB1/Nrf2 signaling pathway, focusing on the role of neutrophils in this process. Furthermore, exploring strategies to enhance the therapeutic efficacy of exosomes derived from mesenchymal stem cells for osteoarthritis treatment is a valuable area of investigation.

## Conclusion

In conclusion, our research offers fresh insights into the pathophysiology and treatment of osteoarthritis. Despite the existing problems and challenges, we could be optimistic about finding more effective treatment strategies in the future due to the advancements in technology and further research, which leads to hope for patients.

This study elucidates how extracellular vesicles derived from mesenchymal stem cells activate the Nrf2 signaling pathway by transporting TGFB1, offering novel insights into the pathophysiological mechanisms underlying osteoarthritis. Drawing on current research, future studies should explore the precise mechanisms underlying the TGFB1/Nrf2 signaling pathway and the role of neutrophils in osteoarthritis progression. As mechanism research progresses, it becomes possible to consider conducting preliminary clinical trials to investigate the actual effects of exosomes derived from mesenchymal stem cells in the treatment of osteoarthritis. Based on the integration of bioinformatics, molecular biology, and clinical medicine, it is essential to delve deeper into comprehensive treatment strategies for osteoarthritis in order to enhance the quality of life for affected patients. The mechanism unraveled in this study could potentially be relevant to other diseases, such as rheumatoid arthritis or inflammatory bowel disease. The potential application of this mechanism should be considered across a broader spectrum of disciplines in the future. This research contributes to the academic community’s increased understanding of osteoarthritis pathogenesis and suggests new avenues for future basic research.

### Electronic supplementary material

Below is the link to the electronic supplementary material.

Supplementary Material 1

Supplementary Material 2

Supplementary Material 3

## Data Availability

No datasets were generated or analysed during the current study.
