# Supplementary figures and images for: Advancing osteoarthritis therapy with GMOCS hydrogel-loaded BMSCs-exos
Source: J Nanobiotechnology. 2024 Aug 19;22:493. doi: 10.1186/s12951-024-02713-z (PMC11334447; doi:10.1186/s12951-024-02713-z)

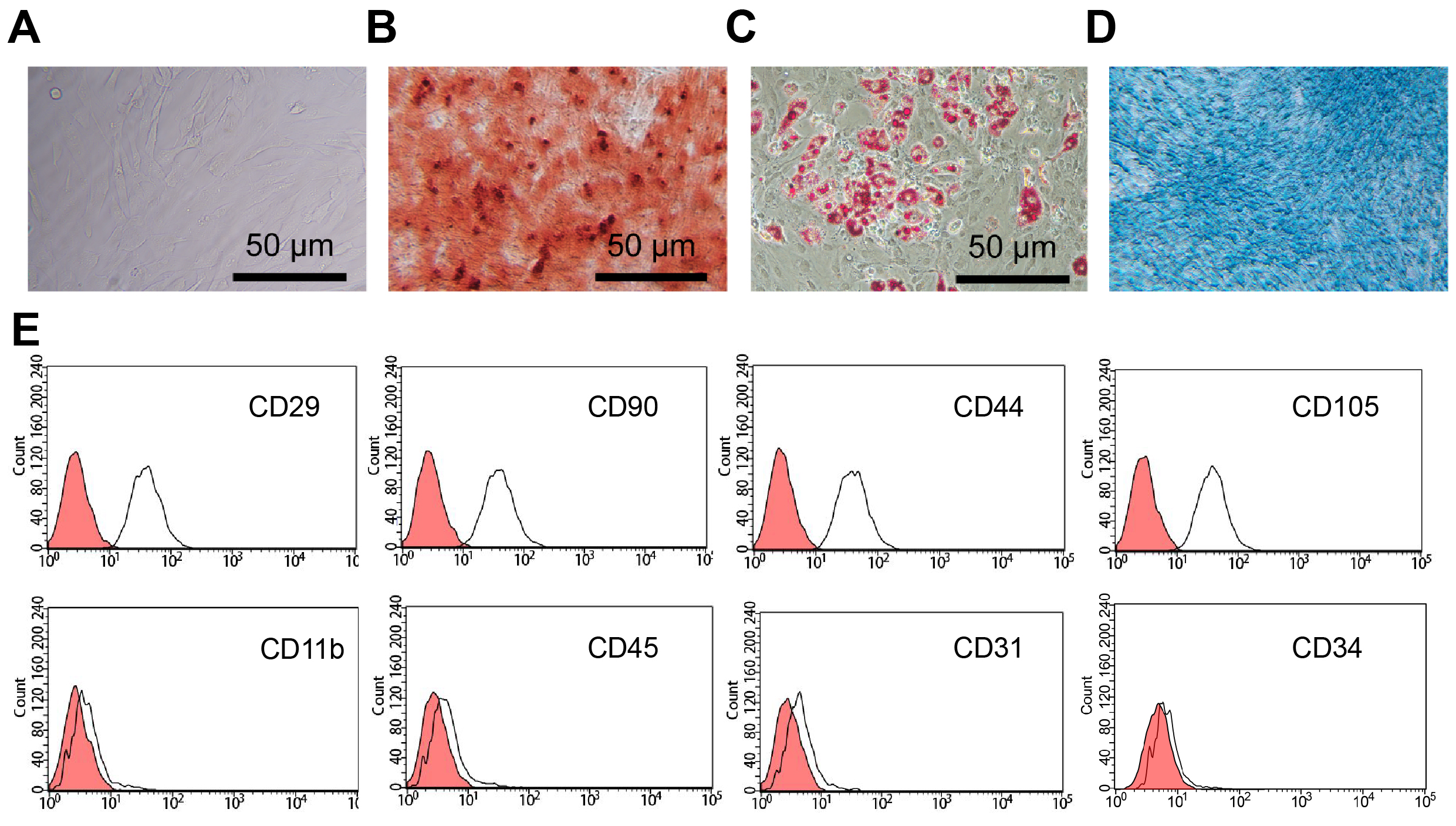

Supplement: Supplementary file 1 — Supplementary Material 1 [file 12951_2024_2713_MOESM1_ESM.jpg]

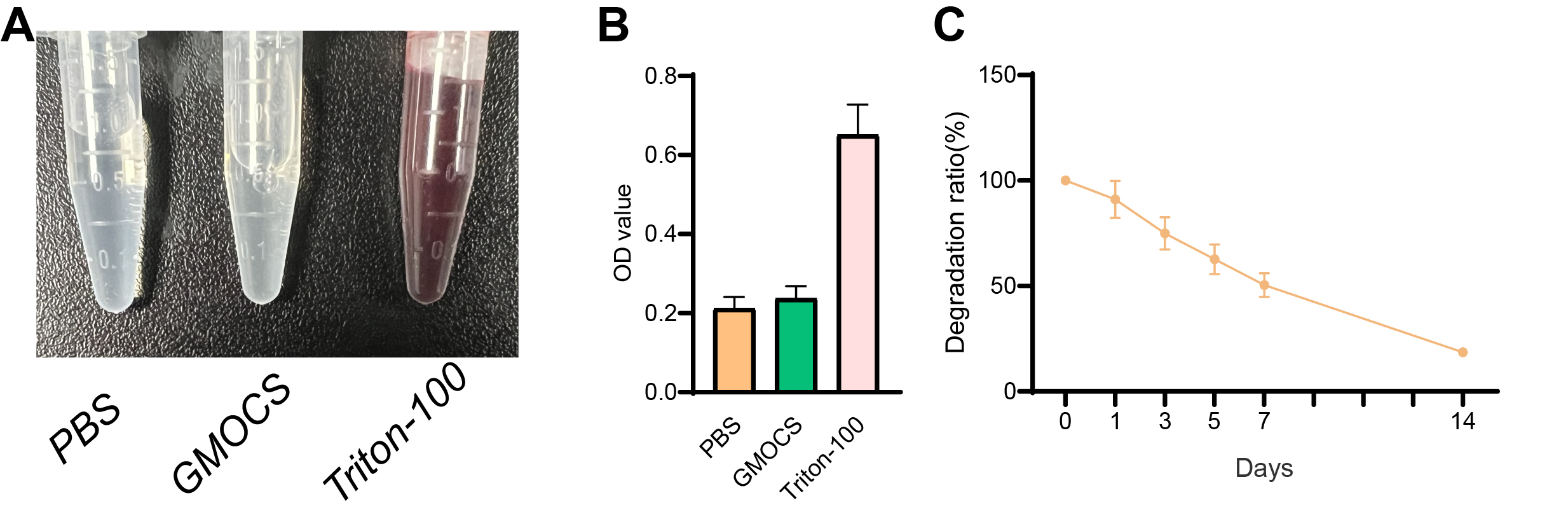

Supplement: Supplementary file 2 — Supplementary Material 2 [file 12951_2024_2713_MOESM2_ESM.jpg]
