# Supplementary material for: Advancing osteoarthritis therapy with GMOCS hydrogel-loaded BMSCs-exos
Source: J Nanobiotechnology. 2024 Aug 19;22:493. doi: 10.1186/s12951-024-02713-z (PMC11334447; doi:10.1186/s12951-024-02713-z)
Supplement: Supplementary file 3 — Supplementary Material 3 [file 12951_2024_2713_MOESM3_ESM.docx]

**Table S1. RT-qPCR primer sequence.**

| Genes | Sequences(5’-3’) |
| --- | --- |
| COL2(Human) | F: GCTCCTGCCGTTTCGCTG |
|  | R: ATTATACCTCTGCCCATCCTGC |
| SOX9(Human) | F: TCTGAACGAGAGCGAGAAGC |
|  | R: CCGTTCTTCACCGACTTCCT |
| MMP13(Human) | F: GCACTTCCCACAGTGCCTAT |
|  | R: AGTTCTTCCCTTGATGGCCG |
| β-actin(Human) | F: GAGAAAATCTGGCACCACACC |
|  | R: GGATAGCACAGCCTGGATAGCAA |
